# Calmodulin regulates Ca_v_3 T-type channels at their gating brake

**DOI:** 10.1074/jbc.M117.807925

**Published:** 2017-09-25

**Authors:** Jean Chemin, Valentina Taiakina, Arnaud Monteil, Michael Piazza, Wendy Guan, Robert F. Stephens, Ashraf Kitmitto, Zhiping P. Pang, Annette C. Dolphin, Edward Perez-Reyes, Thorsten Dieckmann, Joseph Guy Guillemette, J. David Spafford

**Affiliations:** From the ‡ Institut de Génomique Fonctionnelle, CNRS, INSERM, Université de Montpellier, Montpellier F-34094, France,; the Departments of § Chemistry and; ¶ Biology, University of Waterloo, Waterloo, Ontario N2L 3G1, Canada,; the ‖ Division of Cardiovascular Sciences, Faculty of Biology, Medicine and Health, University of Manchester, Manchester M13 9NT, United Kingdom,; the ** Department of Neuroscience and Cell Biology, Rutgers Robert Wood Johnson Medical School, New Brunswick, New Jersey 08901,; the ‡‡ Department of Neuroscience, Physiology and Pharmacology, University College London, London WC1E 6BT, United Kingdom, and; the §§ Department of Pharmacology, University of Virginia, Charlottesville, Virginia 22908

**Keywords:** calcium channel, circular dichroism (CD), cryo-electron microscopy, gating, isothermal titration calorimetry (ITC), nuclear magnetic resonance (NMR), patch clamp, short hairpin RNA (shRNA)

## Abstract

Calcium (Ca_v_1 and Ca_v_2) and sodium channels possess homologous CaM-binding motifs, known as IQ motifs in their C termini, which associate with calmodulin (CaM), a universal calcium sensor. Ca_v_3 T-type channels, which serve as pacemakers of the mammalian brain and heart, lack a C-terminal IQ motif. We illustrate that T-type channels associate with CaM using co-immunoprecipitation experiments and single particle cryo-electron microscopy. We demonstrate that protostome invertebrate (LCa_v_3) and human Ca_v_3.1, Ca_v_3.2, and Ca_v_3.3 T-type channels specifically associate with CaM at helix 2 of the gating brake in the I–II linker of the channels. Isothermal titration calorimetry results revealed that the gating brake and CaM bind each other with high-nanomolar affinity. We show that the gating brake assumes a helical conformation upon binding CaM, with associated conformational changes to both CaM lobes as indicated by amide chemical shifts of the amino acids of CaM in ^1^H-^15^N HSQC NMR spectra. Intact Ca^2+^-binding sites on CaM and an intact gating brake sequence (first 39 amino acids of the I–II linker) were required in Ca_v_3.2 channels to prevent the runaway gating phenotype, a hyperpolarizing shift in voltage sensitivities and faster gating kinetics. We conclude that the presence of high-nanomolar affinity binding sites for CaM at its universal gating brake and its unique form of regulation via the tuning of the voltage range of activity could influence the participation of Ca_v_3 T-type channels in heart and brain rhythms. Our findings may have implications for arrhythmia disorders arising from mutations in the gating brake or CaM.

## Introduction

Calmodulin (CaM)^3^ is a universal resident calcium sensor that promotes a calcium-dependent regulation at a canonical IQ motif of the C-terminal tails of voltage-gated calcium channels (1–5) and sodium channels (6). CaM regulates a rapid and robust inactivation gating of L-type calcium channels (*e.g.* Ca_v_1.2) (7) or skeletal muscle sodium channels (*e.g.* Na_v_1.4) (2) and a facilitation of channel currents in synaptic (*e.g.* Ca_v_2.1) calcium channels (8). The conservation of CaM binding extends to basal Ca_v_1 L-type channels in single-celled eukaryotes, such as ciliate *Paramecium tetraurelia,* which has an extended C terminus that includes a conserved IQ motif (9). CaM mutants of *Paramecium* (10) influence the calcium-dependent inactivation of L-type calcium currents (11, 12) and their control of ciliary beat frequency for swimming and turning behavior (13). CaM binding at the IQ motif and its conserved regulation are consistent with the observed calcium-dependent inactivation (known as CDI) even in expressed cnidarian L-type channel homolog (14). All vertebrate L-type channels display a calcium-dependent inactivation that ranges from minor to very robust, from Ca_v_1.4 to Ca_v_1.1 to Ca_v_1.2 and Ca_v_1.3 channels, respectively (15). A hallmark of the calcium-dependent inactivation in L-type calcium channels is its resilience even in the presence of high-calcium buffering in 10 mm EGTA or BAPTA. This resilience in calcium sensing in the presence of high-calcium buffering is conferred by a secondary CaM-binding site referred to as NSCaTE (N-terminal spatial Ca^2+^-transforming element) specifically contained within the N terminus of invertebrate Ca_v_1 channels (9) and mammalian Ca_v_1.2 and Ca_v_1.3 channels (16, 17). NSCaTE is lacking outside of L-type calcium channels, but the highly conserved C-terminal IQ motif extends to members of the sodium channel family, from Na_v_2 channels in the early branching single-celled eukaryotes before the split of animals and fungi, such as apusozoan, *Thecamonas trahens*, and Na_v_1 channels (18), including cnidarian jellyfish, which are the extant relatives of the likely ancestors containing the first metazoan nervous systems (19, 20). The only member of the calcium and sodium channel superfamily to lack the reported CaM-binding elements (the C-terminal IQ motif and the N-terminal NSCaTE) are Ca_v_3 T-type channels (1, 2, 21).

Here, we show that Cav3 T-type channels possess high-affinity calmodulin binding at the “gating brake,” a helix–loop–helix motif located in the proximal I–II linker of known Ca_v_3 T-type channels in the analogous position where accessory Cavβ subunits regulate Ca_v_1 and Ca_v_2 channel complexes (22). This gating brake governs the low-voltage dependence of T-type channels, and its absence generates a “runaway gating” phenotype (23–26). Mutations that alter the function of the Ca_v_3.2 gating brake have been found in human patients with absence epilepsy (27, 28).

CaM-binding sequences are variable, which can range from a striking likeness to the C-terminal IQ motif from L-type channels, as in the gating brake of the basal metazoan *Trichoplax adherens*, to diverse sequences that generate a nanomolar affinity for CaM binding in protostome invertebrates such as pond snail LCa_v_3 and all human (Ca_v_3.1, Ca_v_3.2, and Ca_v_3.3) channel isoforms. We demonstrate that CaM facilitates the formation of α-helices in gating brake sequences, can pre-associate with Ca_v_3 T-type channels without calcium ions, and its binding involves structural conformational changes in both N- and C-terminal pairs of EF hands in CaM and the gating brake. Dialysis of CaM binding (CaMB) peptides, or co-expression of apo-CaM (CaM_1234_) generates a significant hyper-polarizing shift in voltage sensitivities and faster gating kinetics, consistent with the mutant phenotype of Ca_v_3 T-type channels lacking a gating brake in the I–II linker.

Cav3 T-type channels contribute to pacemaker rhythms, such as the conducting system of the heart (29) and low-threshold calcium potentials (also known as low-threshold spikes), that trigger rhythmic burst firing classically associated with thalamic neurons during non-rapid eye movement sleep and to the spike wave discharge during absence seizures (30, 31). There is also significant evidence for T-type currents participating in “low threshold” neurotransmitter release (32) and in the maintenance of vascular tone (33). Ca_v_3 T-type channels possess a significant “window current” that provides a small but continuous stream of calcium influx through a population of Ca_v_3 T-type channels open at rest (34). This calcium, available through Ca_v_3 T-type channels at rest, is modeled to contribute to cellular proliferation during organ development, to the aberrant proliferation in many cancers (35), and to the hypertrophied condition of the mammalian heart (36). Classically, Ca_v_3 T-type channels are mostly inactivated at rest, and their participation rate is steeply voltage-dependent (37). CaM's regulation of the voltage sensitivities of Ca_v_3 T-type channels at the gating brake thus has dramatic consequences to the participation of Ca_v_3 T-type channels in normal functions, as well as during development and disease.

## Results

### CaM complexes with full-length Ca_v_3 T-type channels

**Figure 1.**
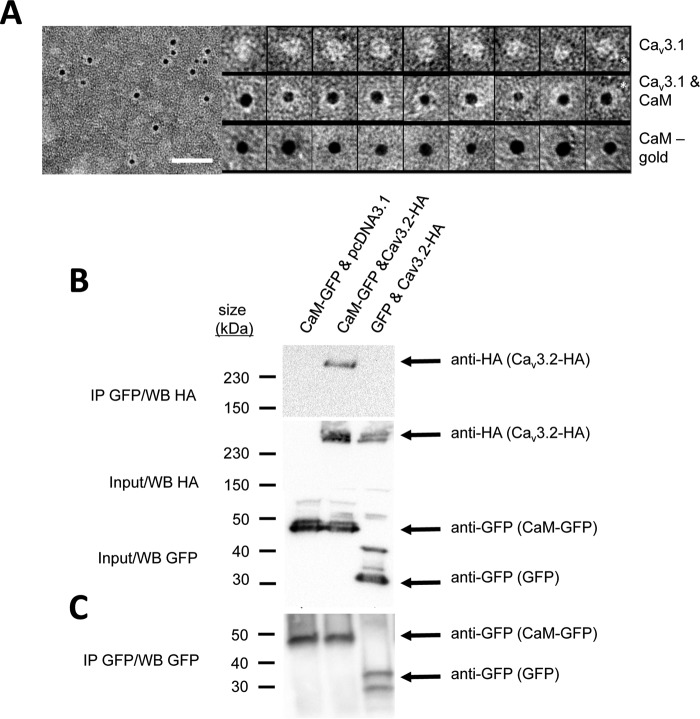
CaM associates with full-length Ca_v_3 T-type channels illustrated by nano-particle cryoelectron microscopy (*A*) and co-immunoprecipitation (*B*). *A,* field of negatively stained (2% w/v uranyl acetate) full-length Ca_v_3.1 in complex with CaM in the presence of 1.5 mm Ca^2+^. *Scale bar* is 50 nm. *Left panel,* CaM has a biotin label that binds streptavidin gold (5 nm). Protein appears *white,* and the *gold* that is electron-dense is *black. Right panel, top row*, montage of purified Ca_v_3.1 particles (*white density*) presenting different views of the channel. *Asterisk* indicates “tail” domain that we have previously determined corresponds to the C terminus (38). *Middle row,* montage of Ca_v_3.1 particles with CaM–biotin bond indicated by the presence of streptavidin gold (*black sphere*). *Bottom row,* control images of CaM–biotin–streptavidin gold particle without Ca_v_3.1. Observed relative differences in Ca_v_3.1 and streptavidin gold particle sizes relate to the orientation and contrast of each Ca_v_3.1 channel particle adhered to the EM support film and the size variation in the streptavidin gold particles (3–6 nm with nominal size of 5 nm, according to the manufacturer). *B,* CaM–GFP bound to anti-GFP magnetic beads associates with hemagglutinin (HA)-tagged Cav3.2 channel (*top panel, middle lane*) as illustrated by HA antibody labeling (259-kDa band) of the Ca_v_3.2 channel co-immunoprecipitant (*IP*) bound to beads. 259-kDa HA-tagged Cav3.2 channel band does not appear as a co-immunoprecipitant in the Western blot without co-expression of pCDNA3.1 plasmid inserts containing HA-tagged Cav3.2 channel (*top panel, left lane*) or without co-expression of CaM–GFP (*top panel, right lane*) in HEK-293T cells. *Middle panel* illustrates anti-HA antibody staining of the 259-kDa HA-tagged Cav3.2 channel of a replicate experiment of input proteins for the Western blot shown in the *top panel* without co-immunoprecipitation. *Bottom panel* illustrates anti-GFP antibody staining of the 44.2-kDa GFP-tagged CaM or GFP alone (27 kDa), in a replicate experiment of input proteins for the Western blot shown in the *top panel* without co-immunoprecipitation. *C,* CaM–GFP (*left two lanes,* 44.2 kDa) and GFP alone (*right lane,* 27 kDa) bound to anti-GFP magnetic beads as illustrated by anti-GFP antibody labeling (259-kDa band) of the Ca_v_3.2 channel co-immunoprecipitant bound to beads. GFP alone generated two bands on the Western blot, which may result from differing post-translational modifications. Co-immunoprecipitation experiments were carried out in 33.3 μm CaCl_2_, pH 7.4. Vector for HEK-293T cell expressed inserts for Western blotting (Cav3.2–HA, EGFP, CaM–pGFP) were contained in pcDNA3.1. Membranes were stained with Ponceau red following protein transfer to evaluate the protein content in each lane. Co-immunoprecipitation experiments were carried out in 33.3 μm CaCl_2_, pH 7.4. Vector for HEK-293T cell expressed inserts for Western blotting (Cav3.2–HA, EGFP, CaM–pGFP) were contained in pcDNA3.1. Membranes were stained with Ponceau red following protein transfer to evaluate the protein content in each lane.

Full-length mammalian Ca_v_3.1 channels were purified from Sf9 insect cells (38) and co-incubated with biotin–CaM complexed to a streptavidin–gold conjugate. Fig. 1*A* (*left panel*) shows a field of purified Ca_v_3.1 (protein appears *white*) coupled to an electron dense (*black*) gold particle, indicating CaM is bound. Individual images illustrating different orientations of the single particles of Ca_v_3.1 channels alone or calmodulin–gold alone and Ca_v_3.1 channels in complex with calmodulin–gold are shown in montages of viewpoints on *top, bottom*, and *middle panels*, respectively, in Fig. 1*A*. The orientation of the channel in some images allows identification of the C terminus, a protruding, finger-like projection from the transmembrane domain of Ca_v_3.1 as highlighted by an asterisk; a feature previously identified through single-particle electron microscopy 3-D reconstruction of Ca_v_3.1 (38). We confirmed the complexing of calmodulin with Ca_v_3 T-type channels as co-immunoprecipitants in Western blottings (see Fig. 1*B*). CaM–GFP fusion proteins transfected and purified from HEK-293T cells and isolated on anti-GFP Sepharose beads were identified in the complex with HA-tagged Ca_v_3.2 channels in Western blottings, labeled with anti-HA antibody, as an ∼259-kDa band (Fig. 1*B*, *middle lane*) in the presence of calcium ions (33.3 μm CaCl_2_). The co-immunoprecipitation of CaM–Ca_v_3.2 channels failed in the absence of co-expressed HA-tagged Ca_v_3.2 channels (Fig. 1*B*, *left lane*) or in the absence of CaM (Fig. 1*B*, *right lane*).

### Ca_v_3 T-type channels possess a predicted high-affinity CaM-binding site at their gating brake in the I–II linker

**Figure 2.**
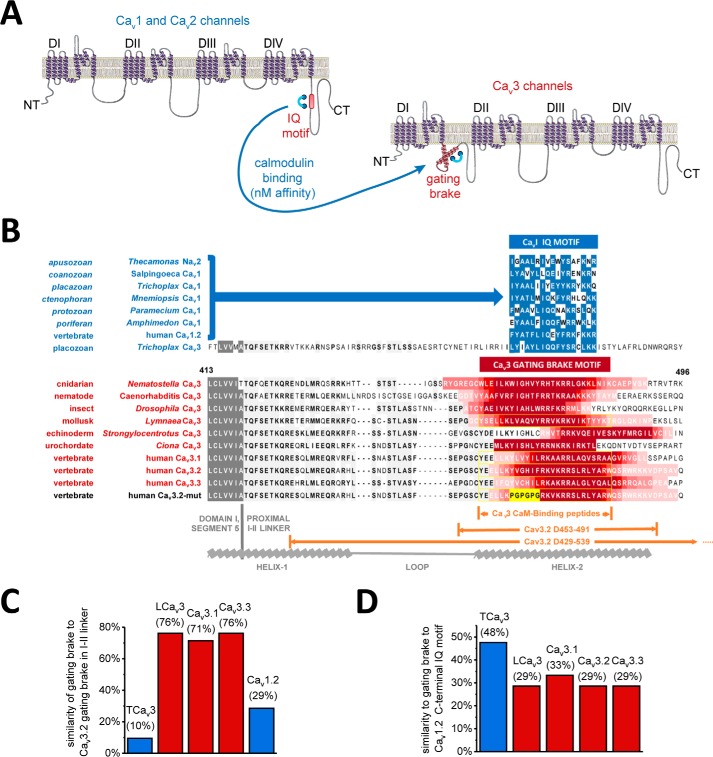
**CaM is predicted to associate with Ca_v_3 T-type channels in helix-2 of the gating brake in the proximal I–II linker.** *A,* illustration of the four domain (DI, DII, DIII, and DIV) × 6 transmembrane helices structure common to sodium channels, calcium channels, and NALCN. Ca_v_1, Ca_v_2, and Na_v_ channels contain a canonical proximal C-terminal IQ motif that possess high-affinity CaM-binding site. A proposed equivalence of the CaM-binding C-terminal IQ motif in Ca_v_3 T-type channels is a helix–loop–helix gating brake motif in the proximal I–II linker that is in the analogous position of β-subunit binding to Ca_v_1 and Ca_v_2 calcium channels. *B,* sequence alignment of T-type channels illustrates a predicted CaM-binding site in helix-2 of the gating brake (illustrated by *red color*) in representative species from cnidarians to the three human genes. CaM-binding site prediction from CaM Target Database (39). Predictions suggested the putative CaMB peptide sequence is cytoplasmic, and helical wheel analysis indicates its amphipathic nature. In the most primitive metazoan with a T-type channel, *Trichoplax* (placozoan), the most basal extant T-type channel in multicellular organisms known possesses a gating brake motif resembling the C-terminal IQ motif shared with other calcium (Ca_v_1 and Ca_v_2) and sodium (Na_v_2 and Na_v_1) channels (illustrated by *blue color*). *Yellow outlined residues* are the sequences for the synthetic CaMB peptides. 39 and 111 amino acid sequence deleted in gating brake deletion mutant, Ca_v_3.2(D^453–491^) and Ca_v_3.2(D^429–539^) are indicated. *C* and *D,* protein similarities among CaMB peptide sequences in the I–II linker, illustrating the high similarity among invertebrate (snail) LCa_v_3 and the human Ca_v_3.*x* homologs (*C*) and the greater similarity of *Trichoplax* Ca_v_3 CaMB peptide sequences to the C-terminal IQ motif of Ca_v_1.2 channels (*D*), rather than other gating brake sequences (*C*).

Ca_v_3 T-type channels lack the C-terminal IQ motif shared among other calcium channels (Ca_v_1 and Ca_v_2) and sodium (Na_v_1 and Na_v_2) channels (1, 2, 21). Because both EM and co-immunoprecipitation experiments indicated that CaM binds to full-length mammalian Ca_v_3.1 and Ca_v_3.2 channels, we therefore next sought to delineate the CaM-binding domain. A unique but ubiquitous feature in Ca_v_3 T-type channels is a helix–loop–helix, gating brake motif (similar in structure to a region in fumarase enzyme) in the proximal I–II linker that is in the analogous position of β subunit binding to Ca_v_1 and Ca_v_2 calcium channels (25). It is within the second helix of the gating brake of the I–II cytoplasmic linker where we have found an analogous region to the C-terminal IQ motif, with nanomolar affinity binding to CaM (Fig. 2*A*). Sequence alignments of Ca_v_3 T-type channels (Fig. 2*B*) indicated a predicted CaM-binding site (illustrated by *red color gradient*) in helix-2 of the gating brake of representative metazoan species from cnidarians to the three human Ca_v_3.1, Ca_v_3.2, and Ca_v_3.3 channels. A CaM-binding site is also predicted in Ca_v_3 T-type channels by the on-line tool, CaM Target Database (39). Predictions suggest that the gating brake sequence is cytoplasmic, and a helical wheel analysis indicates its amphipathic nature.

### Ca_v_3 T-type channel from most basal multicellular animals resembles the C-terminal IQ motif of Ca_v_1, Ca_v_2, Na_v_2, and Na_v_1 channels

The most basal multicellular organism with a Ca_v_3 T-type channel is *T. adherens* (a placozoan) (40), which has a region spanning the gating brake that is unlike other gating brake sequences (Fig. 2*C*, *blue bar*) with a core 17 amino acids that resembles the C-terminal CaM-binding IQ motif of Ca_v_1, Ca_v_2, and Na_v_2 channels (Fig. 2, *C* and *D, blue bars*). The Ca_v_3 T-type channel in the basal multicellular placozoan may represent a structural intermediate involving the positioning of the C-terminal IQ motif in the common ancestor to calcium channels and sodium channels to the proximal I–II linker, before a divergence and appearance of a gating brake helix–loop–helix motif shared among all other metazoan Ca_v_3 T-type channels. Comparatively, the IQ motif of Ca_v_1 L-type channels is mostly identical from microbial eukaryotes to human homologs (9), where the CaM-binding gating brake motif in Ca_v_3 T-type channels is more divergent. The protein similarity of gating brake sequences between protostome invertebrate (*e.g.* pond snail LCa_v_3) and among the human Ca_v_3 isoforms is 71–76% (Fig. 2*C*, *red bars*). Differences in gating brake sequences may reflect a local adaptability of the gating brake among Ca_v_3 T-type channels in different animals.

### Gating brake peptides from snail LCa_v_3 and the three human Ca_v_3.1, Ca_v_3.2, and Ca_v_3.3 T-type channels associate with Ca^2+^–CaM

**Figure 3.**
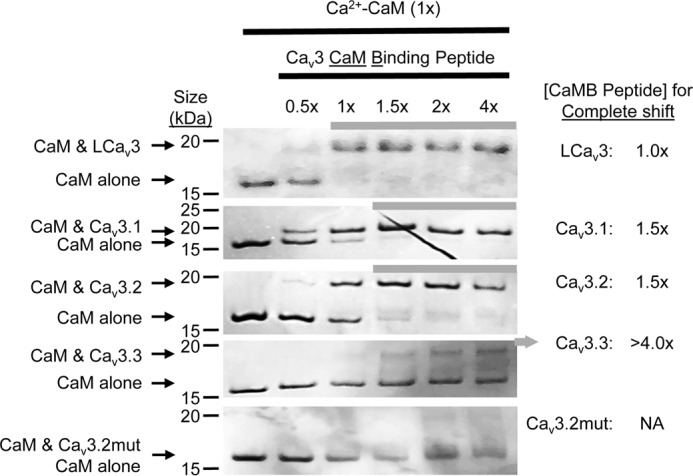
**CaMB peptides from snail LCa_v_3 and the three human Ca_v_3.*x* T-type channels associate with Ca^2+^–CaM.** Gel-mobility shifts with CaM are evident in the presence of 0.1 mm CaCl_2_-containing solution and increasing molar ratios (0.5–4×) of snail LCa_v_3 or human Ca_v_3.1, Ca_v_3.2, and Ca_v_3.3 channel CaMB peptides but not evident in the presence of mutated control Ca_v_3.2 CaMB peptide (Ca_v_3.2mut). *1st lane* in each gel is Ca^2+^–CaM-only control. The rank order of weakest to strongest apparent interaction of Ca^2+^–CaM for peptides: Ca_v_3.3 < Ca_v_3.2 < Ca_v_3.1 < LCa_v_3 correlates with the rank order of calculated binding affinities using ITC (see Fig. 5). Note that Ca_v_3.1 and Ca_v_3.2 peptides both completely displace Ca^2+^–CaM at 1.5× peptide to CaM molar ratio, but Ca_v_3.1 appears to possess a higher affinity based on sub-saturation levels of peptide. Urea attenuates the weaker interacting human Ca_v_3.*x* channels but does not attenuate the higher affinity LCa_v_3 CaMB peptide.

26-mer peptide sequences spanning the gating brake region were synthesized for snail LCa_v_3 and human Ca_v_3.1, Ca_v_3.2, and Ca_v_3.3 channels dubbed “Ca_v_3 CaMB” peptides. In addition, a PGPGPG substituted Ca_v_3.2 channel peptide (Ca_v_3.2mut) was synthesized, which served as a negative control peptide in experiments (see *yellow highlighted sequence* in Fig. 2*B*). CaMB peptides promoted a gel-mobility shift with CaM in the presence of 0.1 mm CaCl_2_-containing solution and increasing molar ratios (0.5 to 4×) of snail LCa_v_3 or human Ca_v_3.1, Ca_v_3.2, and Ca_v_3.3 channel CaMB peptides (Fig. 3). Mutated Ca_v_3.2 CaMB peptide (Ca_v_3.2mut) with the PGPGPG substitution did not promote a gel-mobility shift with CaM (Fig. 3). Ca_v_3.3 peptide has the weakest apparent interaction compared with other Ca_v_3.1 and Ca_v_3.2 peptides, which correlates with its weaker predicted binding affinity (Fig. 2*B*). Presence of urea attenuated the weakest interacting human Ca_v_3.3 channels with CaM without affecting the higher affinity LCa_v_3 gating brake peptide with CaM.

### Gating brake peptides assume a helical conformation upon association with Ca^2+^–CaM

**Figure 4.**
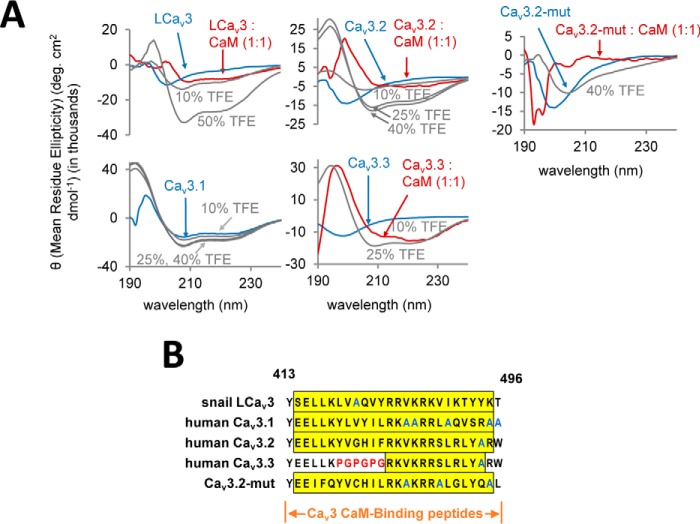
**CaMB peptides from snail Ca_v_3 and human Ca_v_3.*x* T-type channels assume an α-helical structure when bound to Ca^2+^–CaM.** *A,* differential circular dichroism (*CD*) spectra of CaMB peptides co-incubated in the presence of helix-stabilizing agent, TFE (*gray dashed line*), 20 μm CaM (*red line*), or without CaM (*blue line*). 26-mer snail LCa_v_3, human Ca_v_3.2, and Ca_v_3.3 CaMB peptides (sequence, *bottom right*) assume a more helical secondary structure upon addition of CaM and TFE. TFE has a much greater effect on the highest binding affinity peptide, snail LCa_v_3, than the others. Ca_v_3.1 peptide is the only isoform that is α-helical when free in solution, as evidenced by the characteristic negative peaks at 208 and 222 nm. Ca_v_3.2-Gbmut peptide was incapable of adopting an α-helix even at 50% TFE, nor did it appear to interact with CaM. CaM-alone curve was subtracted from each spectra, converted to mean residue ellipticity (θ), and smoothed. For TFE experiments, 50 μm peptide in PBS was used, whereas the baseline was corrected against PBS as background. *B,* gating brake peptide sequences highlighting the alanine-rich Ca_v_3.1 sequence in *blue outline*, and the “PGPGPG” substitution in *red outline* that serves as the Ca_v_3.2 gating brake mutant. The 24 residues of the 26-mer peptides with a *yellow-colored background* are predicted to contain α-helices according to PSIPRED version 3.3 (Bioinformatics Group at University College London) (91).

Differential circular dichroism (CD) spectroscopy of snail LCa_v_3, human Ca_v_3.2, and Ca_v_3.3 gating brake peptides suggest that the peptides assume a more helical secondary structure upon co-incubation with CaM or with helix-stabilizing agent trifluoroethanol (TFE) (Fig. 4*A*). Helical propensity of peptides increases after titrating higher TFE concentrations from 10 to 50% (41, 42). The apparent α-helical formation of LCa_v_3 and Ca_v_3.2 resembles the CD spectral signature with 10% TFE, whereas Ca_v_3.3 resembles more the spectral signature with 25% TFE. Human Ca_v_3.1 is unique among the gating brake peptides in appearing to assume an α-helical conformation when free in solution (without CaM or TFE) (Fig. 4*A*), and this can be explained by its high-native alanine content (Fig. 4*B*). Although the circular dichroism results are suggestive of induction of helical secondary structure in the peptide upon binding CaM, some of the observed ellipticity changes may be induced by tertiary structural changes, not secondary structural ones (43). Mutated peptide (Ca_v_3.2mut) has no α-helical propensity as predicted due to its proline substitutions (Fig. 4, *A* and *B*).

### Gating brake peptides bind with a high nanomolar affinity to Ca^2+^–CaM

**Figure 5.**
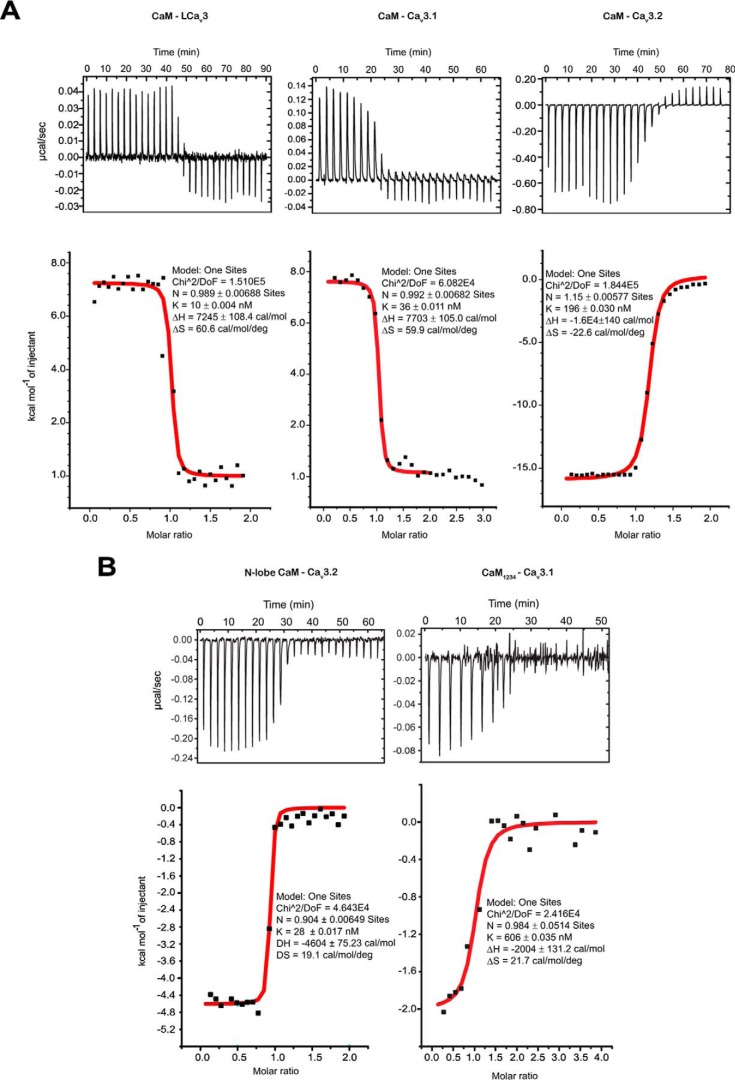
**Thermodynamic basis for CaM-binding peptide interactions with wild-type and mutant CaM.** Raw data traces, isothermal titration calorimetry (*above*), and integrated heats of the measured interaction were fitted with a One Sites model using Malvern MicroCal (ITC200) add-on within Origin software (*below*). *A,* representative interactions with snail LCa_v_3, and human Ca_v_3.1 and Ca_v_3.2 CaMB peptides and wild-type CaM used in the calculation for their 12.4, 42.7, and 187.1 average nanomolar affinities, respectively. *B,* sample interactions with human Ca_v_3.2 and Ca_v_3.1 CaMB peptides and mutant calmodulins, including N-lobe of CaM, and CaM_1234_ used in the calculation for their 31.1 and 543.3 average nanomolar affinities, respectively. Full table of ITC parameters (mean ± S.E., *n* = 3) is illustrated in Fig. 6*A*.

**Figure 6.**
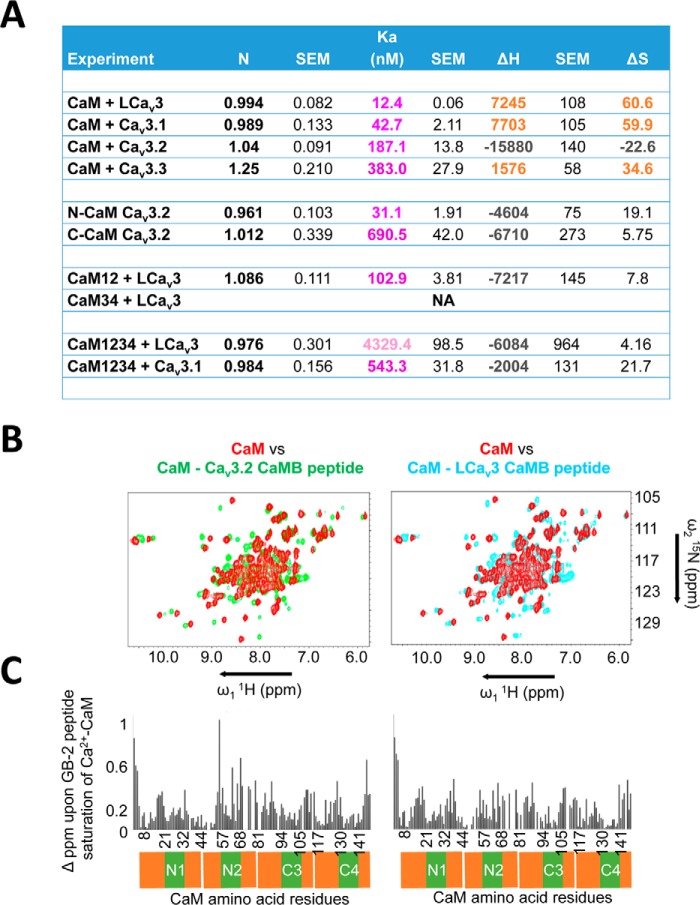
**ITC and NMR analyses indicate high-affinity, nanomolar binding, and associated conformation changes, respectively, upon Ca_v_3 T-type channel CaM-binding peptides to N- and C-terminal lobes of CaM.** *A,* tabulated data of ITC analysis indicates a 1:1 stoichiometry of CaM binding with a 10, 36, 196, and 383 nanomolar affinity for CaM-binding peptides for snail LCa_v_3 and human Ca_v_3.1, Ca_v_3.2, and Ca_v_3.3 channels respectively. All peptide and CaM interactions are endothermic, except Ca_v_3.2 CaMB peptide, which is exothermic. LCa_v_3 CaMB peptide in particular has a complex ITC curve and will bind to CaM with mutated N-terminal Ca^2+^-binding sites (CaM_12_), but not to CaM with mutated C-terminal Ca^2+^-binding sites (CaM_34_). N-CaM and C-CaM are 74-aa constructs (exactly half of the CaM molecule) representing each individual lobe. Both N- and C-CaM associate with Ca_v_3.2 CaMB peptide, albeit a higher affinity for the N-lobe of CaM. CaM with all four Ca^2+^-binding sites mutated in its lobes (apo-CaM, CaM_1234_) still associates with CaM-binding peptides. Table headings: Binding stoichiometry of the interaction between CaM and CaM-binding peptide in solution (*N*), Binding affinity (*K_a_*), enthalpy changes (Δ*H*), entropy changes (Δ*S*). ITC curves were fitted to a one-set-of-sites model with a high degree of fit for *N*, *K_a_*, and Δ*H* values shown for representative experiments in Fig. 5*A*. The parameters for the all peptides were calculated from three replicate experiments. *B,* T-type channel GB peptides promote a conformational change involving both CaM lobes confirmed by amide chemical shifts of CaM's amino acids in ^1^H-^15^N HSQC NMR spectra. Overlay of ^1^H-^15^N HSQC spectra of CaM alone (*red color*) and CaM bound to gating brake peptides from Ca_v_3.2 (*green color*), or snail LCa_v_3 (*cyan color*). *C,* chemical shift differences between CaM and the CaM–Ca_v_3.2 complex and CaM and the CaM–LCa_v_3 complex. *B* and *C* illustrate representative data from one of three replicate experiments.

CaM binds Ca_v_3 CaMB peptides in 0.5 mm CaCl_2_ solution with a 1:1 stoichiometry, at nanomolar affinities that vary from 12, 43, 187, and 383 nm for snail LCa_v_3, Ca_v_3.1, Ca_v_3.2, and Ca_v_3.3 CaMB peptides, respectively (representative data in Fig. 5 and *table of parameters* in Fig. 6*A*). The rank order of measured binding affinities determined in the isothermal titration calorimetry (ITC) (Fig. 6*A*) is consistent with the rank order of concentration of CaMB peptide to CaM ratio required for a saturating gel-mobility shift, 1.0, 1.5, 1.5, and >4.0× for snail LCa_v_3, Ca_v_3.1, Ca_v_3.2, and Ca_v_3.3 CaMB peptides, respectively (Fig. 3). The Ca_v_3.3 CaMB peptide possessed the weakest CaM-binding site of all isoforms as evidenced by the lowest measured affinity with ITC and incomplete gel shift of CaM in native PAGE (Fig. 3). Besides their variable sequence and binding affinity differences for CaM, the Ca_v_3 CaMB peptides vary in their thermodynamic properties during their association with CaM. The association appears to be entropy-driven for LCa_v_3, LCa_v_3.1, and Ca_v_3.3 peptides and enthalpy-driven for Ca_v_3.2 peptide, as illustrated by the latter's negative enthalpy (exothermic) (Figs. 5 and 6*A*).

### Gating brake peptides bind and induce structural changes to both N- and C-terminal lobes of Ca^2+^–CaM and will likely associate with CaM in the absence of Ca^2+^

Mutant CaM_1234_ possess critical Asp → Ala mutations in its EF hands that disable Ca^2+^ binding, creating a mutant that serves as a proxy for apo-CaM (44). The gating brake may serve as a pre-association site for binding CaM in the absence of calcium ions (apo-CaM), with binding of snail LCa_v_3 and human Ca_v_3.1 CaMB peptides with Ca^2+^-binding deficient CaM (CaM_1234_), albeit at an ∼12- and ∼349-fold lower affinity, respectively, compared with wild-type CaM (Figs. 5 and 6*A*). The interactions with CaMB peptides likely involve both lobes of EF hand pairs of CaM, because both N- and C-terminal CaM (74-aa constructs spanning exactly half of the CaM molecule) were able to bind Ca_v_3.2 CaMB peptide, with the N-lobe having a significantly higher affinity (Figs. 5 and 6*A*). LCa_v_3 CaMB peptide will bind to CaM with mutated N-terminal Ca^2+^-binding sites (CaM_12_) but not to CaM with mutated C-terminal Ca^2+^-binding sites (CaM_34_) (Fig. 6*A*). Chemical shift changes observed in the ^1^H-^15^N HSQC NMR spectra of CaM in the presence of both snail LCa_v_3 (*red* to *cyan color shift,* Fig. 6*B*) and human Ca_v_3.2 (*red to green color shift*, Fig. 6*B*) indicate a dramatic conformational rearrangement of both N- and C-terminal lobes of CaM in response to high-affinity binding to gating brake sequences. The widespread and dramatic chemical shift differences in Ca^2+^–CaM alone compared with a CaM–Ca_v_3.2 or CaM–LCa_v_3 CaMB peptide complex is illustrated in Fig. 6*C*.

### Intracellular dialysis of gating brake peptides, but not mutated gating brake peptides, causes a hyperpolarizing shift in voltage dependence and faster gating kinetics in Ca_v_3.2 channels

**Figure 7.**
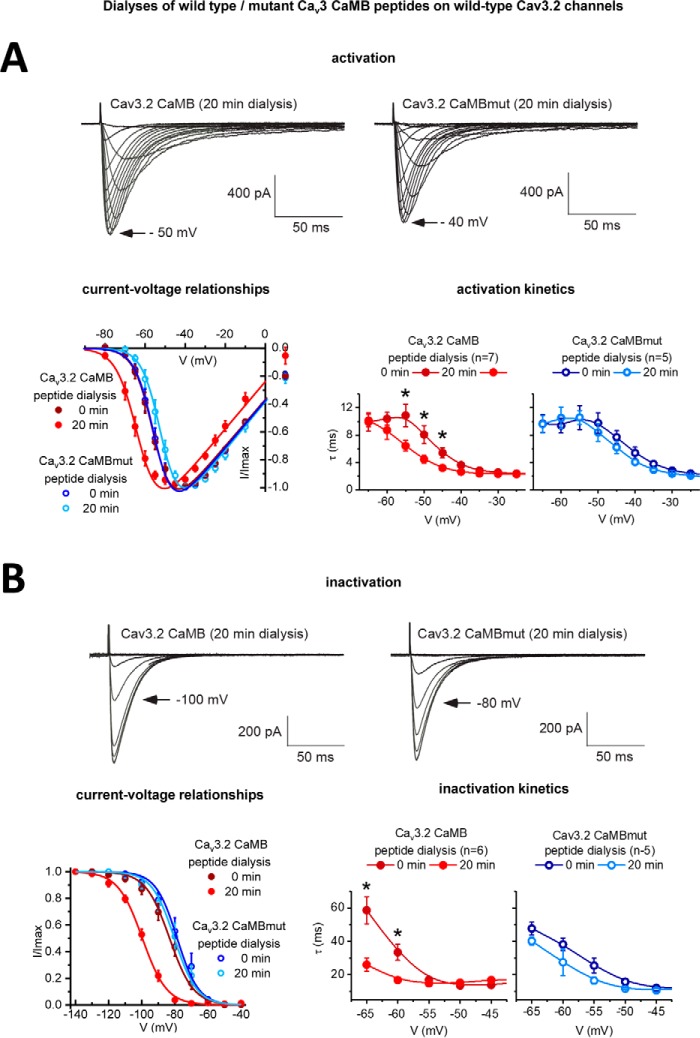
**Intracellular dialysis of 5 μm Ca_v_3 CaMB peptide but not mutated (*CaMBmut*) peptide causes a hyperpolarization shift and faster channel kinetics in Ca_v_3.2 channels.** Transfected Ca_v_3.2 channels in HEK-293T cells were evaluated by whole-cell patch-clamp electrophysiology for activation (*A*) and inactivation (*B*) at start of patch recording (time 0) and after 20 min of intracellular dialysis of 26-mer Ca_v_3 CaM-binding (*CaMB*) peptide or mutated CaM-binding (*CaMBmut*) peptide (peptide sequences in Figs. 2*B* and 4*B*). Representative current traces after 20-min dialyses are shown. Calcium currents were measured for their current-voltage relationships and τ mono-exponential fits for the kinetics of activation (*A*) and inactivation (*B*). Activation and steady-state inactivation curves were created with peak currents generated from a step depolarization from −110 to −80 to −10 mV and to −30 mV from holding potentials ranging from −140 to −40 mV, respectively. Statistical significance (*p* < 0.05) using a non-parametric Wilcoxon matched-pairs signed rank test for the kinetic data is shown by * measured before and after equilibration of the intracellular dialysis of Ca_v_3 CaMB peptide or mutated CaM binding (CaMBmut) peptide. *n* values for current-voltage relationship Ca_v_3 CaMB peptide (*n* = 7) and CaMBmut (*n* = 6). *n* values for inactivation Ca_v_3 CaMB peptide (*n* = 5) and CaMBmut (*n* = 6).

**Figure 8.**
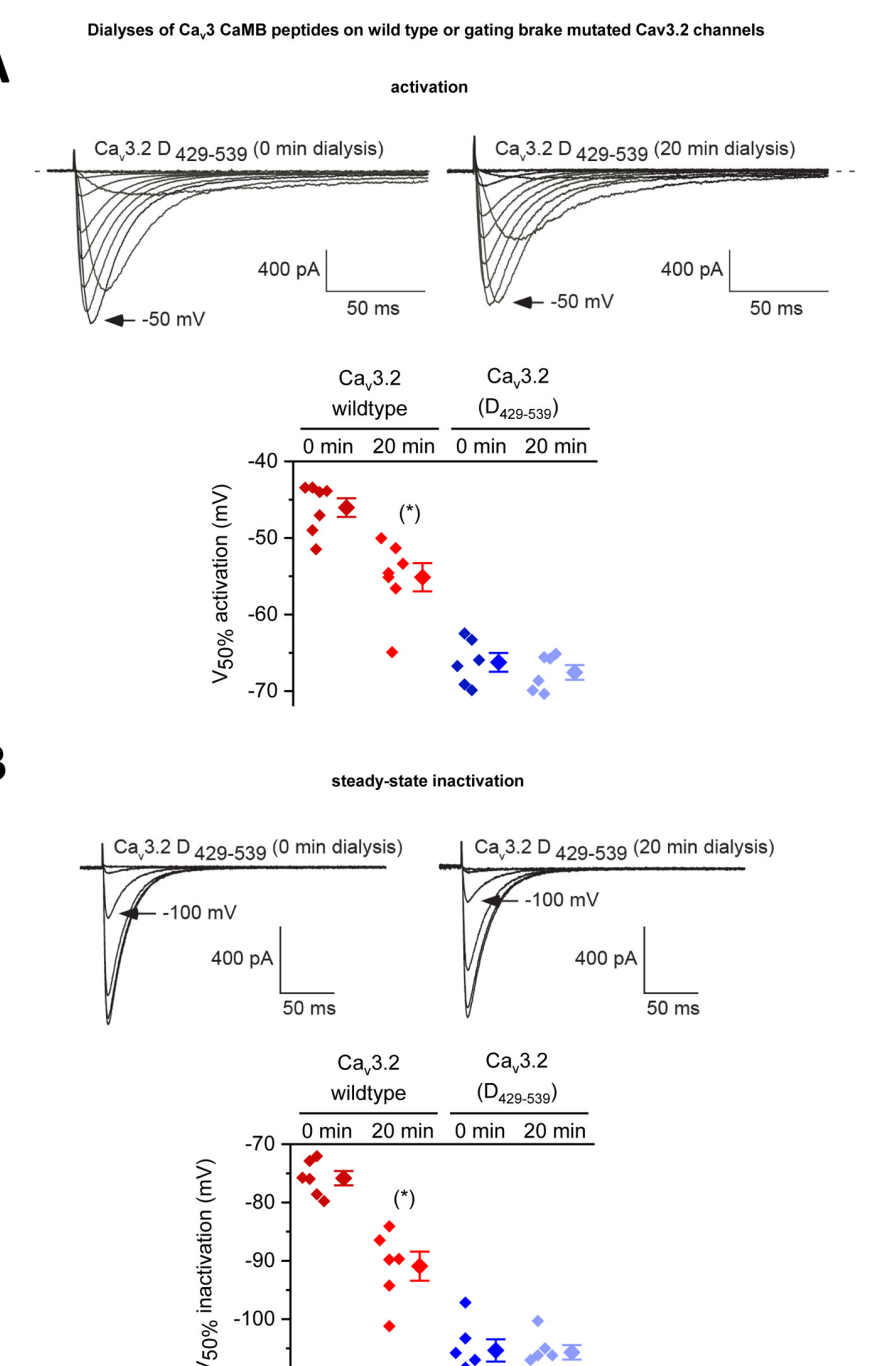
**Intracellular dialysis of 5 μm Ca_v_3 CaMB peptides cause a hyperpolarizing shift in voltage dependence but only with Ca_v_3.2 channels containing an intact gating brake.** Scatterplot (*left*) with mean ± S.E. (*right*) values of the voltages of 50% activation (*A*) and steady-state inactivation (*B*) taken from Boltzmann fits of activation and steady-state inactivation curves for wild-type Ca_v_3.2 channels and Ca_v_3.2 channels with a deleted gating brake (deleted sequence from 429 to 539, see Fig. 2*A*). Representative current traces at time 0 min and after 20-min dialysis of Ca_v_3.2 CaMB peptide are shown. Dialysis of CaMB peptide (after 20 min) generates a hyperpolarizing shift in voltage dependence of wild-type Ca_v_3.2 channels, but it does not promote additional hyperpolarizing shifts in voltage dependence on the Ca_v_3.2 channels that are highly hyperpolarizing shifted after gating brake deletion. The statistical significance after dialyses of CaMB peptide in Ca_v_3.2 channels containing an intact gating brake is *p* = 0.0156 (*) and *p* = 0.0313 (*) for *V*_50%_ activation and *V*_50%_ inactivation, respectively, in a Wilcoxon matched-pairs signed rank test.

**Figure 9.**
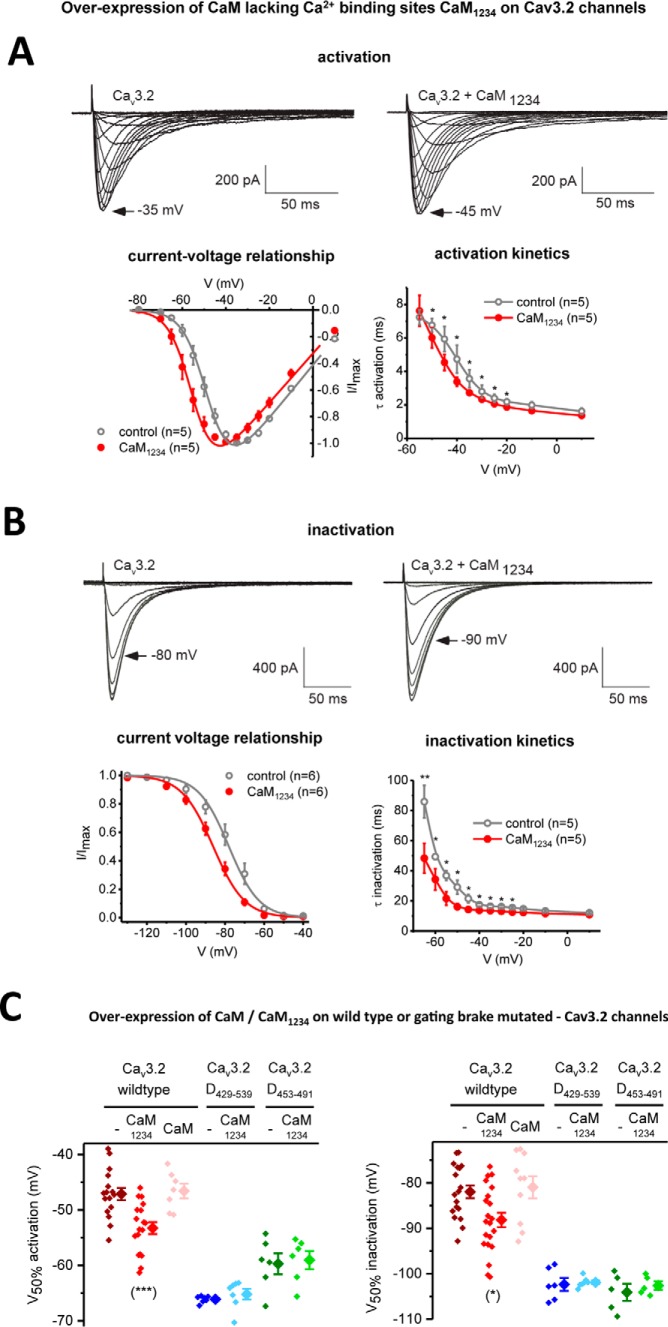
**Overexpression of apo-CaM/CaM_1234_ (Ca^2+^-binding deficient CaM) causes a hyperpolarizing shift in voltage sensitivities and faster channel kinetics in Ca_v_3.2 channels only in the presence of an intact gating brake in Ca_v_3.2.** *A* and *B,* Ca_v_3.2 channels with and without co-expressed CaM_1234_ were measured for their current-voltage relationships and τ for mono-exponential fits of the kinetics of activation (*A*) and inactivation (*B*). Representative current traces are shown. Results are presented as mean ± S.E., and *n* = the number of cells. Statistical analyses were first performed with the Student's *t* test or with one-way ANOVA combined with a Tukey post-test for multiple comparisons (*, *p* < 0.05; **, *p* < 0.01; ***, *p* < 0.001). *C,* scatter plot (*left*) with mean ± S.E. (*right*) values illustrate the 50% activation and inactivation values taken from Boltzmann fits of activation and steady-state inactivation curves of Ca_v_3.2 channels alone (−), and Ca_v_3.2 channels with co-expressed apo-calmodulin (CaM_1234_) or calmodulin (*CaM*). Voltage responses to co-expressed apo-calmodulin (CaM_1234_) are also illustrated for Ca_v_3.2 deletion mutants spanning sequences 429–539 or 453–491, lacking the 111 or 39 amino acids, respectively, flanking the gating brake in the I–II linker. The region of deletion mutants spanning the gating brake of Ca_v_3.2 channels are illustrated in Fig. 2*B*. The statistical significance of CaM_1234_ overexpression compared with wild-type conditions was *p* = 0.0028 (***) and *p* = 0.0308 (*) for *V*_50%_ activation and *V*_50%_ inactivation, respectively, using a Kruskal-Wallis test followed by a Dunn's multiple comparisons test. The effect of CaM_1234_ overexpression compared with wild-type conditions for Ca_v_3.2 deletion mutants (D^429–539^ or D^453–491^) were non-significant using a Mann-Whitney test.

We tested whether the observed nanomolar affinity of CaM for the gating brake governs the unique gating properties of Ca_v_3 T-type channels such as its low voltage of activation. We challenged cells expressing Ca_v_3.2 T-type channels with 5 μm Ca_v_3.2 CaMB peptides pre-loaded in the intracellular pipette, and we evaluated the change of gating behavior in whole-cell patch-clamp recording of transfected Ca_v_3.2 T-type channels in HEK-293T cells, before and after equilibration of the intracellular dialysis of gating brake peptide (after 15–20 min) (Fig. 7). We conducted parallel control experiments with the intracellular dialysis of mutant gating brake peptide (Ca_v_3.2 CaMBmut) (mutant sequence in Figs. 2*B* and 4*B*). The Ca_v_3 CaMB peptide challenge to Ca_v_3.2 channels causes a phenotype change, which resembles the 33- or 111-amino acid deletion mutant phenotype of Ca_v_3 channels lacking the gating brake, Ca_v_3.2(D^453–491^) and Ca_v_3.2(D^429–539^) (23), respectively (Fig. 7). The gating brake is so named because it is considered a unique regulator of Ca_v_3 T-type channels, maintaining them in the closed state at resting membrane potentials (23–26), where disruptions of the gating brake cause the voltage threshold for activation and inactivation gating to be shifted 20–30 mV in the hyperpolarizing direction (*e.g.* see Figs. 8 and 9*B*) (23, 25, 26, 45), with dramatically accelerated activation and inactivation kinetics (23–26). Activation curves are shifted to negative potentials by an average of 8 mV (a 50% activation voltage change from −55.28 ± 1.33 to −63.10 ± 1.29 mV) after dialysis of Ca_v_3.2 CaMB peptide, whereas no corresponding voltage shifts are observed with mutated CaMB peptides (Ca_v_3.2 CaMBmut) (Fig. 7*A*). Steady-state inactivation curves of HEK-293T cell-transfected Ca_v_3.2 channels are also shifted toward negative potentials by an average of 16 mV (a 50% inactivation voltage change from −83.19 ± 2.35 mV to −99.62 ± 1.37 mV) after 20 min of dialysis with Ca_v_3.2 CaMB peptide but not after dialysis with mutated peptide (Ca_v_3.2 CaMBmut)-treated cells (Figs. 7*B* and 8*B*). Intracellular dialysis of Ca_v_3.2 CaMB peptide also confers a significant speeding up of channel activation and inactivation kinetics, compared with Ca_v_3.2 channels dialyzed with mutated peptide (Ca_v_3.2 CaMBmut). This is quantified as significant reduction in the τ for exponential fits of the kinetics of activation (Fig. 7*A*) and inactivation (Fig. 7*B*) in the presence of Ca_v_3.2 CaMB peptide.

### Dialyzed gating brake peptides specifically target the gating brake of Ca_v_3.2 channels

We observed that the intracellular dialysis of Ca_v_3.2GB peptide does not confer additional hyperpolarizing shifts in voltage sensitivity of activation (Fig. 8*A*) or steady-state inactivation (Fig. 8*B*) in Ca_v_3.2 channels possessing the gating brake deletion Ca_v_3.2(D^429–539^). These observations are consistent with the specific targeting of Ca_v_3.2 CaMB peptide to helix-2 of the gating brake region in the I–II linker, a region that possesses a nanomolar affinity for CaM to promote the observed hyperpolarizing shifts in voltage sensitivities of Ca_v_3 T-type channels.

### Results of overexpression experiments with mutated CaM (CaM_1234_) strongly suggest that CaM regulates Ca_v_3.2 channels at the gating brake

ITC measurements suggest that the gating brake associates with both N- and C-terminal Ca^2+^-binding EF hand pairs. CaM binds to the gating brake in the absence of viable calcium-binding sites (CaM_1234_). This CaM association for Ca_v_3.1 and LCa_v_3 CaMB peptide in the absence of calcium is at an ∼12- or ∼349-fold reduced affinity, respectively, compared with wild-type CaM (Fig. 6*A*). We tested whether calcium occupancy in CaM lobes is required to prevent the gating brake phenotype. We overexpressed a plasmid construct coding for CaM_1234_ to compete with native CaM (46). The CaM_1234_ overexpression construct included the bicistronic marker mRFP (red color fluorescent protein) (46) to be used as a visual estimate of the expected level of CaM_1234_ overexpression, after mercury lamp excitation with the epifluorescence-inverted microscope used for patch-clamp recording. The CaM_1234_ overexpression construct also contained two shRNA sequences to knock down CaM (*CALM*_1,2,3_ genes) (46–48). The shRNA knockdown sequences is reported to be 70% complete in disabling native CaM expression (46–48). However, knockdown was not effective under our experimental conditions, as we observed no significant physiological consequence after transfection of the shRNA CaM knockdown alone. The combined construct of shRNA knockdown of wild-type CaM and overexpression of CaM_1234_ had a dominant effect, compared with the overexpression of a similar construct with wild-type CaM (Fig. 9). CaM_1234_ causes a hyperpolarizing shift in voltage dependence for activation and inactivation and more rapid gating kinetics (Fig. 9), resembling the phenotype after saturating CaM-binding sites with dialyzed gating brake peptides (Figs. 7 and 8) and resembling the runaway gating phenotype of T-type channels after deletion or mutation of their gating brake sequences (Fig. 8, *A* and *B*) (23–26). The hyperpolarizing voltage shift conferred by CaM_1234_ appear to require association with an intact gating brake, because additional hyper-polarizing shifts in voltage dependence are lacking in Ca_v_3 channels containing deletion of 39 and 111 amino acids spanning the gating brake, Ca_v_3.2(D^453–491^) and Ca_v_3.2(D^429–539^), respectively (Fig. 9*B*) (23).

## Discussion

### Ca^2+^–CaM readily complexes with full-length Ca_v_3 T-type channels in vitro

We show here that the two most common isoforms of Ca_v_3 T-type channels expressed in both the mammalian brain and heart (Ca_v_3.1 and Ca_v_3.2) (49), in their full-length configurations, complex with Ca^2+^–CaM *in vitro*. We illustrate using electron microscopy that purified full-length Ca_v_3.1 binds calcium–CaM, data supported by co-immunoprecipitation experiments. A recent study by Lee *et al.* reported that a rat Ca_v_3.3 splice variant co-immunoprecipitates with CaM (50), adding support for a universality in CaM associating with all Ca_v_3 T-type channels, in the same manner as CaM serves as a ubiquitous resident calcium sensor at the C-terminal IQ tails of Ca_v_1 and Ca_v_2 calcium channels (1–5) and Na_v_1 sodium channels (6). Ca_v_3 T-type channels (like all eukaryotic calcium and sodium channels), are large proteins (∼250 kDa) that do not readily express well enough for the purposes of extensive *in vitro* biochemical analyses. We chose candidate peptide fragments based on *in silico* prediction for resolving finer details of the CaM regulation of Ca_v_3 T-type channels. Examination of candidate peptides is the standard approach used for other CaM targets (51).

### All known Ca_v_3 T-type channels have a predicted high-affinity CaM-binding site in helix2 of the helix–loop–helix of the gating brake in the I–II cytoplasmic linker

Ca_v_3 channels lack the canonical C-terminal IQ motif (FILV)Q*XXX*(RK)G*XXX*(RK)*XX*(FILVWY) of Ca_v_1 L-type Ca^2+^ channels or (FILV)Q*XXX*(RK)*XXXXXXXX* Na_v_1 sodium channels for CaM association (51). One of the criteria for a potential CaM-binding motif is its omnipresence from microbial eukaryotes to humans such as the IQ motif within different Ca_v_1 and Na_v_ channels (9). This criterion was satisfied with the predicted CaM association in the second helix of the helix–loop–helix motif forming the Ca_v_3 gating brake in the I–II linker of almost all known Ca_v_3 channels. All 26-mer gating brake sequences are similar (*e.g.* 71–76% between pond snail and human Ca_v_3 isoforms) but are also completely lacking the string of identical and canonical amino acids conserved in the IQ motif in different Ca_v_1 and Na_v_ channels. Interestingly, the most basal of multicellular organisms, the placozoan species *T. adherens*, possess a Ca_v_3 T-type channel (40) but lack an identifiable gating brake motif (only 10% similarity to pond snail and human Ca_v_3 isoforms). In its place, the *Trichoplax* channel sequence is similar to the IQ motifs of L-type calcium channels (48% to the human Ca_v_1.2). It is highly suggestive that the earliest eukaryotic Ca_v_3 T-type channels had a CaM-binding IQ motif shared with other Ca_v_ and Na_v_ channels, before its divergence and establishment of its unique identity in Ca_v_3 T-type channels, as the gating brake.

### Conformational changes in both Ca^2+^–CaM and CaMB peptides are observed upon their high nanomolar affinity association

Nanomolar and specific CaM binding to Ca_v_3 CaMB peptides are corroborated in the ability of Ca^2+^–CaM to increasingly associate with Ca_v_3 CaMB peptides corresponding to increasing concentrations of the Ca_v_3 CaMB peptides illustrated by gel-mobility-shift assays. Ca_v_3 CaMB peptides appear to undergo dramatic conformational changes (*i.e.* increasing α-felicity) on association with Ca^2+^–CaM illustrated by circular dichroism. Although ellipticity changes in the circular dichroism likely involve the induction of α-helices in the peptide upon binding CaM, some of the changes may be induced by tertiary structural changes in the proteins themselves (43). The association of the Ca_v_3 CaMB peptides with CaM generates dramatic conformational changes in Ca^2+^–CaM, illustrated by amide chemical shifts of CaM's amino acids in ^1^H-^15^N HSQC NMR spectra spanning both N- and C-terminal lobes of CaM. Thermodynamic properties measured within the isothermal cell of the Ca^2+^–CaM complexing with Ca_v_3 CaMB peptides illustrate that Ca^2+^–CaM and Ca_v_3 CaMB peptides associate in a 1:1 stoichiometry (*N* value) at a high nanomolar association constant (*K_a_*).

### Highest affinity association of Ca^2+^–CaM CaMB peptides requires both lobes of CaM and their Ca^2+^-binding sites

Further evidence of the specificity of the Ca_v_3 CaMB peptides to Ca^2+^–CaM is in the requirement of intact Ca^2+^–CaM for high nanomolar affinity binding. We show that the N-lobes and C-lobes of CaM alone will associate with Ca_v_3 CaMB peptides, but at a lower measurable affinity compared with both CaM lobes. The high-affinity of CaM for Ca_v_3 CaMB peptides requires intact Ca^2+^-binding sites for CaM, as evidenced by the greatly weakened affinity of CaM in the CaM_1234_ configuration for the Ca_v_3 CaMB peptides.

### Different sequences for CaMB peptides from sample invertebrate LCa_v_3 and mammalian Ca_v_3 channels possess a high nanomolar affinity and similar binding properties in complexing with Ca^2+^–CaM

Differing sequences of CaMB peptides for snail LCa_v_3 and the three human Ca_v_3 gene isoforms are 71–76% similar to one another and possess similar binding properties with Ca^2+^–CaM. Snail LCa_v_3 and the different human Ca_v_3 isoforms of the CaMB peptides associate with a nanomolar affinity and in a 1:1 stoichiometry with Ca^2+^–CaM. All CaMB peptides assume a greater α-helicity upon association of Ca^2+^–CaM, except the alanine-rich Ca_v_3.1 isoform, and both snail LCa_v_3 and mammalian Ca_v_3.2 CaMB peptides generate similar and large conformational changes to both N- and C-lobes of CaM as measured by the amide chemical shifts of CaM's amino acids in ^1^H-^15^N HSQC NMR spectra. Both snail LCa_v_3 and mammalian Ca_v_3.2 CaMB peptides also associate with Ca^2+^–CaM at a much weakened affinity when Ca^2+^-binding sites are mutated in CaM.

### Differing sequences of CaMB peptides differ in their relative affinities for Ca^2+^–CaM

The sequence differences between the Ca_v_3 CaMB peptides contributed to significant differences in binding affinities, which varied from rank order of highest to lowest binding affinities of LCa_v_3 > Ca_v_3.1 > Ca_v_3.2 > Ca_v_3.3 of 12, 43, 187, and 383 nm, respectively. The rank order of binding affinities measured by the thermodynamic changes resulting from the 1:1 complexing of Ca_v_3 CaMB peptides and Ca^2+^–CaM measured in the isothermal cell is also the same observed rank order of the fold concentration of Ca_v_3 CaMB peptides required for saturation of Ca^2+^–CaM with Ca_v_3 CaMB peptides for a complete gel-mobility shift.

### Nanomolar affinities of Ca_v_3 CaMB peptides are approximately equivalent to the measured high affinities of the IQ motif of Ca_v_1.2 and Ca_v_2.1 channels and greater than the affinity of Ca^2+^–CaM for Na_v_1.5 channels

Our measured binding affinities of Ca_v_3 CaMB peptides for N- and C-lobes of Ca^2+^–CaM (in nm) of 31 ± 1.9 and 690 ± 42, respectively, is in the relative range of nanomolar affinities for CaM as the C-terminal IQ domain of Ca_v_1.2 of 57.6 ± 35.5 and 2.63 ± 0.07 (52) and the C-terminal IQ domain of Ca_v_2.1 of 51 ± 20 and 4.32 ± 0.39 (53). The higher nanomolar binding affinity of the Ca_v_3.2 channel CaMB peptides for N- and C-lobes of Ca^2+^–CaM is greater than NSCaTE, a secondary CaM-binding site of Ca_v_1.2 channels (in nm): 650 ± 60 and 1330 ± 300 (16), respectively. Ca_v_3.2 channel CaMB peptides are also much higher affinity than the micromolar binding affinity range for the N- and C-lobes of Ca^2+^–CaM of Na_v_1.5 sodium channel fragments (in nm), such as its C-terminal IQ domain of 2170 ± 400 and 6130 ± 900 (6) and the III–IV linker of 19,270 ± 940 and >500,000, respectively (54).

### Phenotype after dialysis of Ca_v_3 CaMB peptides corresponds to immobilizing the gating brake in the I–II linker

Our biochemical studies reflect that Ca^2+^–CaM associates with helix-2 of the gating brake in both invertebrate and all mammalian isoforms of Ca_v_3 channels, in a high nanomolar affinity with binding characteristics that is reminiscent of the canonical binding of Ca^2+^-CaM to the proximal C-terminal IQ motif of Ca_v_1 and Ca_v_2 channels. We then tracked the consequences of introducing the high-affinity CaMB peptide in expressed Ca_v_3.2 channels in real time, by comparing the biophysical changes to the Ca_v_3.2 channels from the start of a stabilized patch-clamp recording (0 min) and after complete dialysis of 5 μm CaMB peptides from our patch pipette (15–20 min). Biochemically, we observed that equivalent mutated CaMB peptide did not associate with Ca^2+^–CaM, so it served as a control to compare the effects of the CaMB peptide. We observed ∼8- and ∼16-mV hyperpolarization shifts in activation and inactivation curves, respectively, as a result of CaMB peptide dialysis. Faster channel kinetics were also observed, which are a likely a consequence of the hyperpolarizing shift, because corresponding kinetic changes are usually associated with shifts in voltage sensitivities within Cav3 T-type channels (37). The target for the CaMB peptide effect was the gating brake, as we observed no additional hyperpolarizing shift in voltage sensitivities after CaMB peptide dialysis in Ca_v_3.2 channel mutants lacking the gating brake. We carried out the complete set of electrophysiology experiments using mammalian Ca_v_3.2 because of the availability of gating brake mutants. We will carry out future analyses to confirm the hyperpolarizing shifts in voltage sensitivities in response to CaMB peptide dialyses are shared among other Ca_v_3 T-type channels, including invertebrate LCa_v_3, all of which have a common nanomolar affinity for CaM binding to the gating brake.

### Overexpression of Ca^2+^-binding deficient CaM (i.e. CaM_1234_) reveals that CaMB peptides likely compete for CaM binding at the gating brake in the I–II linker of Ca_v_3 channels

Involvement of CaM regulation of ion channels has been supported by introducing a Ca^2+^-insensitive mutant form of CaM (CaM_1234_), which possess point mutations within both pairs of EF hands in the N- and C-lobes of CaM to eliminate Ca^2+^ binding (44). It is believed that CaM pre-associates onto its target in the apo-CaM form (lacking Ca^2+^ occupied in its EF hands) (55–57). CaMB peptides have a measurable affinity with CaM_1234_, albeit at levels that are ∼12- and ∼349-fold lower compared with wild-type CaM, for Ca_v_3.1 and LCa_v_3 CaMB peptides. Apo-CaM bound to its channel target is considered a necessary antecedent to the subsequent regulation of CaM by Ca^2+^. Overexpression of the mutant apo-CaM, which is incapable of binding Ca^2+^, is thus considered a competitive inhibitor to silence the effects of Ca^2+^–CaM. For example, the dominant-negative effects of CaM_1234_ overexpression have been used to illustrate the importance of Ca^2+^–CaM in the gating of small-conductance Ca^2+^-activated K^+^ channels (44), and the necessity of Ca^2+^–CaM in the Ca^2+^-dependent inactivation of Ca_v_1 L-type channels (58, 59) as well as the calcium-dependent facilitation of Ca_v_2 channels (60). We observe that CaM_1234_ causes a modest (6.7 and 7.2 mV) hyperpolarizing shift in activation and inactivation curves of Ca_v_3.2 channels, respectively, compared with wild-type CaM overexpression. The likely target for the CaM_1234_ effect is the gating brake, as we observed no additional CaM_1234_ causing hyperpolarizing shifts on Ca_v_3.2 channels lacking the gating brake.

### Combined biochemistry and electrophysiology data highly suggest that Ca^2+^–CaM is a high-affinity resident sensor at the gating brake of Ca_v_3 channels

To summarize our data, we illustrate that CaM_1234_, considered as a substitute for apo-CaM (CaM in the absence of calcium), associates with helix-2 of the gating brake at a low micromolar affinity. Upon binding Ca^2+^, CaMB peptides and CaM both undergo dramatic conformational changes, leading to CaM's tighter association at the gating brake at a higher nanomolar affinity. Our data suggest that Ca^2+^–CaM is behaving as a similar high-affinity and resident sensor at the gating brake in the I–II linker of Ca_v_3 T-type channels, in both sample invertebrate and the three human gene isoforms, in a universal manner similar to the calcium–CaM regulation on the IQ motif containing C-terminal tails of Ca_v_1 and Ca_v_2 calcium channels (1–5) and Na_v_1 sodium channels (6). Introducing CaMB peptides or CaM_1234_ competitively inhibits Ca^2+^–CaM at the gating brake of Ca_v_3.2 channels, generating a runaway gating phenotype, with hyperpolarized shifts in voltage sensitivities that are even greater than observed after deletion of the brake from Ca_v_3 channels. Because the introduced CaMB peptides or CaM_1234_ have no effect on Ca_v_3.2 channels with a missing gating brake, it suggests that the primary target for Ca^2+^–CaM is the gating brake of Ca_v_3 channels. We did not observe the complexing of full-length Ca_v_3.1 and Ca_v_3.2 channels with CaM *in vitro* in the absence of calcium ions, but we suspect that it may relate to the structural requirements for calcium ions in recapitulating the native behavior of full-sized, Ca_v_3 channels *in vitro*, the sensitivities of our assays, and the technical challenges in working with whole ion channel proteins of such large sizes (∼259 kDa) *in vitro*.

### Ca_v_3.3 exhibits a similar calcium-dependent CaM regulation as other calcium and sodium channels

Consistent with our findings here, Lee *et al.* (50) report a hyperpolarizing shift in activation and inactivation curves of Ca_v_3.3 channels and faster inactivation kinetics, under conditions where intracellular free calcium is at a higher (1 μm) but not lower (27 nm) calculated concentration. Lee *et al.* (50) also observe a faster inactivation kinetics in Ca_v_3.3 channels associated with external calcium compared with barium as the charge carrier. The much faster inactivating mammalian T-type channels, Ca_v_3.1 and Ca_v_3.2, do not appear to exhibit the same calcium-dependent change in biophysical properties as Ca_v_3.3 channels but possess a more pronounced voltage-dependent inactivation (61). Lee *et al.* (50) illustrate that rat Ca_v_3.3 channels possess a unique C-terminal sequence that associates with Ca^2+^–CaM, conferring the unique CaM regulation observed for Ca_v_3.3 channels.

### Observed variability of the Ca^2+^-dependent regulation within Ca_v_3 channels is consistent with the variability in Ca^2+^–CaM binding within the Ca_v_1 L-type channels

A suggested hypothesis is that all Ca_v_3 channels possess a central, high-affinity Ca^2+^–CaM binding at the gating brake in the I–II linker, with additional modulatory, intracellular Ca^2+^–CaM-binding sites elsewhere, which can confer novel attributes, such as the Ca^2+^–CaM regulation at the C terminus for Ca_v_3.3 channels (50). A highly customizable form of Ca^2+^–CaM regulation is observed within the “IQ motif centric” calcium (Ca_v_1 and Ca_v_2) and sodium (Na_v_1) channels. The preoccupancy of CaM at the primary IQ motif in the absence of Ca^2+^ ions is one form of regulation. The specialized Ca_v_1.4 homolog localized in vertebrate photoreceptors possesses a highly conserved IQ motif but exhibits almost no calcium-dependent inactivation compared with closely-related Ca_v_1.2 channels. Ca_v_1.4 possess a distal C-terminal auto-inhibitory domain, which effectively competes for apo-CaM for binding, thus preventing Ca^2+^–CaM regulation at Ca_v_1.4's conserved proximal C-terminal IQ motif (62). Ca_v_1.3 possess a similar auto-inhibitory domain in the distal C terminus generated through alternative splicing, which competes with apo-CaM (63, 64).

Another source of variability is in the semi-autonomy and cooperativity among CaM's pairs of Ca^2+^-binding EF hands in the N- and C-lobes, each of which is capable of conferring unique attributes. For example, the Ca^2+^–CaM-binding site NSCaTE shared within the N terminus of invertebrate Ca_v_1 channels (9) and mammalian Ca_v_1.2 and Ca_v_1.3 channels (16, 17) interacts specifically with CaM's N-lobes, conferring a capacity for more global Ca^2+^-sensing and buffer-resistant calcium-dependent inactivation in classical L-type channels. The C-lobe of CaM is occupied with the IQ motif, imparting a local, more rapid, and spatially restricted calcium-sensing of Ca^2+^ ions (16, 17).

### Observed variability of the Ca^2+^-dependent regulation within Ca_v_3 channels is consistent with the variability in Ca^2+^–CaM binding within the Ca_v_2 synaptic Ca^2+^ channels and Na^+^ channels

The bipartite nature of CaM's N- and C-lobes is also evident in the synaptic Ca_v_2 channel family, where Ca^2+^ regulation manifests as a rapid, calcium-dependent facilitation of the calcium current triggered at CaM's C-lobe and a slower-developing calcium-dependent inactivation imparted by CaM's N-lobe (60, 65).

One confounding issue is a potential interplay between calcium- and voltage-dependent properties, such as the consequences of a Ca^2+^–CaM-binding site identified in the III–IV linker of Na_v_1.5 sodium channels, which is also the region responsible for the fast voltage-dependent inactivation within sodium channels (6, 54, 66). The contributions of both calcium- and voltage-dependent properties can obscure the typical U-shaped calcium-dependent inactivation (39).

### Technical challenges in measuring the Ca^2+^ occupancy of CaM in vivo

Observations *in vitro* often do not correlate to those gathered within different cell types too, where different factors influence CaM's effects within the cellular environment. For example, when CaM has many high-affinity targets within a cell, it has been reported that CaM availability is likely to be limiting in many cell types (67, 68). In such cases, CaM regulation may be unavailable because individual CaMs may be preoccupied at different high-affinity cellular targets. There is also likely competition among differing calcium-sensing proteins. A good example is calcium-binding protein expressed in the vertebrate nervous system, whose expression can antagonize CaM-dependent regulation of Ca_v_1 L-type channels (69).

The last confounding issue with studies involving CaM is in the technical challenge in modeling calcium availability for CaM regulation. It is challenging to precisely measure native calcium-buffering conditions because they are variable within a particular cell or among different cell types. Ion channel studies usually involve intracellular dialysis in the whole-cell patch-clamp configuration of intracellular calcium chelators (*e.g.* EGTA and BAPTA), which impose artificial calcium-buffering conditions, whose calcium affinity and on-off rates are not known in comparison with the native buffering conditions *in vivo*.

### Universal “calmodulation” among sodium and Ca^2+^ channels also involves Ca_v_3 channels

Ca_v_3 channels are the least well studied of the calcium or sodium channels, which means that Ca_v_3 channels are interpreted through the lens of what is understood for other calcium and sodium channels (37). A universal IQ centric calmodulation of sodium and calcium channels has more variations than commonality by the many interactions that fine-tune the Ca^2+^–CaM regulation outside of the primary C-terminal IQ motif. So it should not be surprising that a functional Ca^2+^–CaM regulation is only evident within one of three mammalian Ca_v_3 channels in preliminary analyses, and lacking evidence so far within Ca_v_3.1 and Ca_v_3.2 channels (50). Despite possessing proximal C-terminal Ca^2+^–CaM binding in the IQ motif and the III–IV linker, cardiac muscle Na_v_1.5 channels possess no obvious Ca^2+^–CaM regulation, whereas skeletal muscle Na_v_1.4 has a robust calcium-dependent inactivation (2, 70, 71). A deeper molecular analysis uncovered, for example, a Ca^2+^–CaM regulation of classical L-type channels within Ca_v_1.4 channels, after deletion of its C-terminal auto-inhibitory domain (62).

### Potential implications for Ca_v_3 channels possessing Ca^2+^–CaM regulation at the gating brake

Ca_v_3 channels are equally distant structurally to both calcium and sodium channels (37), and it should come as no surprise that its Ca^2+^–CaM regulation is also strikingly different, where the equivalence to the C-terminally positioned IQ motif is transposed onto a different cytoplasmic region. The gating brake is a unique element within all Ca_v_3 T-type channels, which tunes the unique low voltage sensitivity of this channel class (72). The gating brake is a position that overlaps with the Alpha1 Interaction Site of Ca_v_1 and Ca_v_2 calcium channels where accessory Ca_v_β subunits regulate their expression levels and biophysical properties (25). Ca_v_3 T-type channels lack equivalency of the high-nanomolar affinity Ca_v_β subunit-binding site within Ca_v_1 and Ca_v_2 calcium channels (73). It is possible to confer Ca_v_β subunit regulation onto Ca_v_3 T-type channels after transplanting the I–II linker from Ca_v_2 channels (45). But in their native state, Ca_v_3 T-type channels possess remarkably similar properties as their *in vivo* counterparts without requirement of co-expressed accessory subunits in invertebrates (74) and mammals (45, 75). Ca_v_β subunits will boost the level of expression of invertebrate (76) and mammalian (75) Ca_v_3 T-type channels, but this is considered to be the result of indirect and generalized consequences of overexpressing a multifunctional, scaffolding, and regulatory protein, such as the Ca_v_β subunit *in vitro* (77).

With a lack of equivalent accessory subunit interactions within Ca_v_3 channels, opportunities are created for a Ca^2+^–CaM regulation within the gating brake of the I–II linker. The gating brake is placed in the I–II loop, just downstream of a stiff α-helical linker extending from the segment 6 of domain II, which contributes to pore opening (72). Affixing Ca^2+^–CaM to the I–II loop may have structural implications because the I–II loop is immobilized at both ends compared with the C-terminal tails containing the IQ motif of sodium and calcium channels, which is linked only a single end. A more proximate location of the gating brake to the intracellular pore mouth may also have implications, contributing to a unique Ca^2+^–CaM regulation within Ca_v_3 channels.

### Potential importance of Ca^2+^–CaM regulation of Ca_v_3 channels for human physiology and disease

The unique high-affinity binding and regulation of CaM at the gating brake of Ca_v_3 T-type channels has dramatic potential consequences, because of the gating brake's role as a primary regulator of the voltage sensitivity of Ca_v_3 T-type channels. Ca_v_3 T-type channels are unique among sodium and calcium channels by the low voltage range of activation that is close to typical resting membrane potentials. CaM at the gating brake is perfectly positioned in Ca_v_3 channels for fine-tuning the channels' responsiveness to voltage, providing a means to govern the capacity of Ca_v_3 T-type channels to facilitate pacemaker rhythms where T-type channels have established roles, such as the thalamo-cortical circuits of the brain (where Ca_v_3 T-type channel expression is highest) and the conducting system in the heart (49). Human mutations in the gating brake are associated with arrhythmic vulnerabilities such as epilepsy (27, 28) or autism spectrum disorder (78), and it is the platform where Ca^2+^ and CaM can modulate the low-voltage gating behavior in T-type channels, in a manner that is unique from the regulation of CaM universally at the IQ motif in the C-terminal tails of Ca_v_1 and Ca_v_2 calcium channels (1–5) and Na_v_1 sodium channels (6).

### Outside of Ca^2+^–CaM regulation, Ca_v_3 channels are very different from other calcium and sodium channels

Ca_v_3 channels uniquely different Ca^2+^–CaM regulation goes alongside its many observed differences from other sodium and calcium channels. This includes their ion selectivity, which can range from mostly calcium-conducting (Ca_v_3.1 and Ca_v_3.2), equally sodium- and calcium-conducting (Ca_v_3.3) and as sodium-conducting as Na_v_1 sodium channels (protostome invertebrate Ca_v_3 channel isoforms).

We have shown that most non-vertebrate Ca_v_3 T-type channels have alternative splicing of the L5 extracellular turret in domain II, which converts the mostly calcium-conducting T-type channel into a highly sodium-conducting isoform, whose expression in the invertebrate heart serves as the primary sodium current (74). The plasticity of invertebrate Ca_v_3 channels in assuming calcium- or sodium-conducting pore configurations is to expand their niche in the absence of Na_v_1 channel genes expressed outside of the nervous systems of many invertebrates, and in the many species of non-vertebrates completely lacking any Na_v_1 channel in their genomes (nematodes, echinoderms, hemichordates, and parasitic flatworms) (74). It is an interesting question to address how Ca^2+^–CaM regulation varies when Na^+^ is the primary conducting ion rather than Ca^2+^ through differing invertebrate Ca_v_3 channel isoforms.

### Potential role for calcium sensing at the gating brake is in influencing calcium ion selectivity within Ca_v_3 channels

We have shown that the Ca_v_3 channels are endowed with ∼2-fold increase in the number of cysteines in L5 and L6 extracellular turrets, contributing to uniquely folded extracellular scaffolds (79). In particular, alternative turrets with unique cysteine configurations in both DII L5 and DIV L6 regulate the degree of sodium and calcium conductances through invertebrate Ca_v_3 channels.^4^ Deletion of these unique L5 cysteines in domain II, alters drug affinity, divalent cation block with Ni^2+^ and Zn^2+^, and also increasing the apparent pore size, allowing for the accommodation of a simultaneous flow of both monovalent and divalent cations.^5^ The simultaneous flow of differing divalent and monovalent ions may only be possible within Ca_v_3 T-type channels, given its likely wider, more accommodating EEDD selectivity filter compared with other Ca_v_1 and Ca_v_2 calcium channels with an EEEE selectivity filter (49). One of the possible implications of a high-affinity calcium sensor, such as CaM near the internal mouth of the pore, is in its potential in influencing calcium ion conductance. We observe an apparent simultaneous flow of monovalent and divalent ions through Ca_v_3 channels containing mutated cysteines in the domain II, L5 extracellular loop, which is only possible if the divalent cation alongside the monovalent (*e.g.* Na^+^) is either Sr^2+^ or Ba^2+^ but not Ca^2+^.^5^ A high-affinity calcium sensor, like CaM at the pore mouth, may contribute to the explanation for the observations that Ca^2+^ appears as the dominant and preferred divalent ion carrier through the Ca_v_3 channel pore, even when the channel is highly sodium-conducting.^5^

## Experimental procedures

### Expression of Ca_v_3 constructs

The cDNAs encoding for Ca_v_3.2 were previously characterized (61) as well as hemagglutinin (HA) epitope-tagged Ca_v_3 subunits expressed using pcDNA 3.1 vector (23, 75). We illustrate how to maintain HEK-293T cell lines and introduce calcium channel expressible plasmids for transient transfection, expression, and whole-cell patch-clamp recording in an on-line video (JoVE) format (82). HEK-293T cells were cultured as described previously (23, 75). For optimal transfection, cells were plated at 50–70% confluence. Cells were transfected using the JetPEI transfection reagent (QBiogene) according to the manufacturer's protocol. In all conditions, corresponding empty plasmids were used to adjust the quantity of the transfected material (1 μg of each plasmid/35-mm dish). Gating brake deletion mutants for Ca_v_3.2, D^429–539^, and D^453–491^ were used previously (23).

### Expression of CaM, CaM mutant, and CaM knockdown constructs

We have used human CaM and associated CaM mutants that we have described before (9), CaM(1–44) and partial CaMs: N-CaM(1–74), C-CaM(75–148), and CaMs that simulate CaM binding in the absence of calcium, by substitution of glutamate residues in the two calcium-binding sites in the N-terminal lobe (CaM_12_) or C-terminal lobe (CaM_34_), or mutation of all four calcium-binding sites (CaM_1234_) of CaM. CaM–GFP used for Western blotting is a construct consisting of GFP fused to the C terminus of CaM expressed in pcDNA3.1 (Cell Signaling Technology) (83). EGFP expression vector was obtained by subcloning EGFP from pEGFP-C1 (BD Biosciences) into the multiple cloning site of pcDNA3.1. The CaM_1234_ overexpression construct contained bicistronic marker mRFP (to generate a red color) (46) to distinguish from the channel expression with bicistronic EGFP (to generate a green color). The CaM_1234_ overexpression construct also containing CaM shRNA knockdown includes two short hairpin sequences described above to knock down CaM (*Calm*_1,2,3_ genes) (46–48). The shRNA knockdown sequences is reported to be 70% complete in disabling of native CaM expression (46–48), but this knockdown was insufficient for our purposes, as we observed no significant physiological consequence after transfection of the shRNA CaM knockdown alone.

### Synthetic peptide experiments

We purified bacterially expressed His-tagged CaM (CaM) and measured *in vitro* association with 26-mer synthetic peptides (BioBasic) spanning the gating brake of human Ca_v_3.*x*, Ca_v_3.2GBmut, and snail LCa_v_3 in gel-shift mobility assays, differential circular dichroism, and isothermal titration calorimetry. We followed protocols that we have described previously in an analysis of CaM binding with snail or human L-type calcium channel peptides in the presence of 0.1 or 0.5 mm CaCl_2_-containing solution (9).

### CaM protein expression for biochemistry experiments

Rat CaM was purified using phenyl-Sepharose 6 Fast-Flow (High Sub) (GE Healthcare), packed to ∼10 ml volume, by gravity flow at 4 °C. Codon-optimized CaM was cloned in pET9d, a T7 *Escherichia coli* expression vector. CaM was transformed into BL21 DE3 *E. coli* and grown at 37 °C and 200 rpm to OD of 0.4–0.6 (for Luria Broth, LB) or 0.5–0.9 (for SuperBroth, SB), at which point samples were induced by 1 mm added isopropyl β-d-1-thiogalactopyranoside and grown for another 4 (for SB) to 6 (for LB) h. Robust expression of soluble CaM was confirmed by SDS-PAGE. Cells were collected by centrifugation at ∼4000 × *g*, then lysed in high-salt buffer (0.1 m Tris-Cl, pH 7.5, 0.5 m NaCl, 0.5 m ammonium sulfate, 1 mm DTT) using an Avestin Emulsiflex homogenizer at 15,000–20,000 p.s.i. with jacketed water cooling, through which the cells were passed ∼3× on average. The cell lysate was clarified by centrifugation at 20,000 rpm in an SS-34 rotor (∼50,000 × *g*) at 4 °C for 30 min. To the cleared lysate, CaCl_2_ was added to a final concentration of 10 mm. The lysate was then applied to the gravity phenyl-Sepharose column equilibrated with the same high salt buffer (and 1 mm CaCl_2_). The column was washed with high-salt buffer. CaM was then eluted using 10 mm Tris-Cl and EDTA solution, pH 7.5. The highest fractions were pooled, concentrated, and applied to the preparation grade Superdex 75 16/60 column (GE Healthcare) on an AKTA FPLC system. Typical injection volume was 1 ml, and the gel filtration buffer (50 mm Tris-Cl, pH 7.5, 150 mm NaCl, 1 mm CaCl_2_) was set to flow at 1 ml/min. Wild-type CaM reproducibly elutes at ∼68 ml on this column. Elution peak width is dependent on the amount of CaM present; routinely up to 10 1-ml fractions were obtained of concentrations 200–400 μm; these were pooled and concentrated using the spin concentrators from Sartorius or Amicon (10,000 molecular weight cutoff). Extinction coefficient of 3006 m^−1^ cm^−1^ at *A*_276_ was used (66), and the final protein stock was aliquoted to 500 μl and stored at −80 °C.

### Purification of Ca^2+^-deficient CaM proteins

The interaction of CaM with hydrophobic interaction chromatography media depends on its exposure of the hydrophobic residues upon Ca^2+^ binding. The phenyl-Sepharose method is not nearly as efficient for the purification of CaM_12_, CaM_34_, or CaM_1234_. So for Ca^2+^-deficient CaM proteins, we used a combination of ion-exchange chromatography and subsequent gel filtration for purification. A Q-Sepharose (quaternary ammonium) 6-ml column (GE Healthcare) was used for ion exchange. Lysis and low-salt buffer consisted of 5 mm BisTris, pH 6.0, with no Ca^2+^ or EDTA. Lysis buffer also had DTT added to 1 mm final concentration to maintain CaM's methionines in the reduced state. CaM_12_, CaM_34_, and CaM_1234_ (in pET28a vector) were expressed and harvested in same manner as wild-type CaM. Cells were lysed using the Avestin Emulsiflex homogenizer (>15,000 p.s.i.), clarified by centrifugation at ∼ 50,000 × *g,* and applied to the column (ion exchange was done using the AKTA FPLC system, same as for gel-filtration chromatography). The column was then briefly washed with low-salt buffer (5–10 column volumes), and a shallow salt gradient was applied (100 mm NaCl over 10 column volumes). Subsequently, the column proteins were eluted with a steep gradient (1.6 m NaCl over 2 column volumes, held for 2 more column volumes), after which the column was re-equilibrated or cleaned. The resulting fractions were analyzed by SDS-PAGE and read for absorbance levels at 278 nm. Best candidates were pooled and concentrated using YM-10 spin columns (Millipore) and further purified by gel-filtration chromatography. Purity of the CaM proteins was confirmed by SDS-PAGE and ESI-MS (electrospray ionization-mass spectrometry), and their concentrations were determined using the modified Bradford assay (84) (with wild-type CaM as the standard), aliquoted, and stored in gel filtration buffer at −80 °C.

### Bioinformatic analyses of peptide sequences

Protein parameters (pI and molecular weight) were determined using the prediction tool in ExPaSy (http://web.expasy.org/protparam/),^6^ and the aggregation propensity was determined using the on-line software at Centre for Genomic Regulation (http://tango.crg.es/).^6^ All phylogenetic alignments were done using variations of the BLAST tool on the GenBank^TM^ web site. The CaM-binding site prediction database (http://calcium.uhnres.utoronto.ca/ctdb/contacts/index.htm)^6^ was used to search for other possible “hits” in the channel ORFs (39) and to design optimal peptide sequences for synthesis.

### Native Trp fluorescence

Steady-state measurements were obtained using a Photon Technologies International Quantamaster Fluorimeter (London, Ontario, Canada) in a 50-μl 1-mm fluorescence cuvette (Hellma) at room temperature. The same buffer was used as for gel filtration and ITC experiments (50 mm Tris-Cl, pH 7.5, 150 mm NaCl) with either 1 mm CaCl_2_ or 10 mm EDTA; buffer was used as a baseline for subtraction. To the cuvette containing the starting peptide solution (25 μm), increasing amounts of CaM were added (in increments corresponding to ∼2.5 μm). Stepwise mission scans from 300 to 400 nm using a 280-nm excitation wavelength and 1-nm slit width (averaging over 1s each) were performed.

### Dansyl-CaM fluorescence

Dansyl (5-(dimethylamino) naphthalene-1-sulfonyl chloride) is an amine-reactive fluorescent dye. Dansyl-CaM was prepared as described previously (85). CaM (1 mg/ml) was transferred into 10 mm NaHCO_3_, 1 mm EDTA, pH 10.0, at 4 °C. 30 μl of 6 mm dansyl-chloride (1.5 mol/mol of CaM) in DMSO was added to 2 ml of CaM, with stirring. After incubation for 12 h at 4 °C, the mixture was first dialyzed against 500 volumes of 150 mm NaCl, 1 mm EDTA, 20 mm Tris-HCl, pH 7.5, at 4 °C, and then dialyzed against 500 volumes of water. Labeling yields were determined from absorbance spectra using the ϵ320 of 3,400 m^−1^ cm^−1^ and were compared with protein concentrations determined using the Bradford method with wild-type CaM used as the protein standard (86) ESI-MS was used to confirm successful dansyl-labeling of CaM protein. The concentration of dansyl-CaM in all experiments was 2 μm. Steady-state fluorescence was performed in a similar manner as the Trp experiments, except that a newer Fluorolog 3-22 (Horiba Scientific, Ltd.) fluorimeter was used, and the buffer used was 10 mm HEPES, pH 7.0, with supplemented 0.1 mm CaCl_2_.

### Differential circular dichroism

CD measurements were performed using a Jasco-715 spectropolarimeter (Jasco Instruments, Easton, MD), using the following parameters: 250–190-nm range, 50 nm/min speed, with 1-s response time, 1-nm bandwidth, and 100-millidegree sensitivity at room temperature. 16–25 accumulations were gathered depending on signal quality. Recordings were made in a 1-mm cuvette, in 10 mm sodium phosphate buffer with 0.1 mm added CaCl_2_, and used as baseline, which was subtracted from each recording. For CaM and CaMB peptide spectra, the same solution of 10 μm CaM was scanned before stock (1 mm) peptide was added in incremental amounts before being scanned again. The resulting spectra were subtracted (*e.g.* CaM and CaMB peptide-CaM alone) to extract the spectrum corresponding to the change associated with adding the peptide. Every recording was an average of at least 16 accumulations and subsequently corrected for protein concentration (converted to units of mean residue ellipticity). These were smoothed and exported into ASCII format for analysis with Excel and either SOMCD or Dichroweb K2d algorithm (http://dichroweb.cryst.bbk.ac.uk)^6^ (92, 93). TFE recordings were performed using pure 2,2,2-trifluoroethanol (Sigma) at 10, 25, and 50% after thorough pre-mixing and incubation with peptides in the phosphate buffer, baseline-corrected against the same TFE/buffer solution without peptide. LCa_v_3 and Ca_v_3.2 CaMB peptides were contained in PBS buffer (10 mm sodium phosphate, pH 7.3, 137 mm NaCl, 2 mm KH_2_PO_4_, and 2.7 mm KCl). Ca_v_3.1, Ca_v_3.3, CaMB peptides were characterized in low ionic strength buffers due to severe aggregation issues in PBS. Low ionic strength buffer for Ca_v_3.1 and Ca_v_3.3 CaMB peptides was composed of 10 mm HEPES, pH 7. A 0.1-cm quartz cuvette (Hellma) was used for CD measurements. CaM-binding experiments were performed by adding increments of stock (1 mm) CaMB peptide to the same cuvette containing 10 μm CaM (300 μl). Subsequent scans would then have the CaM-only trace subtracted and corrected for total peptide concentration, smoothed, and converted to mean residue ellipticity units.

### Gel mobility-shift assays

Gel assays were run in native conditions with 4 m urea, containing 15% acrylamide separating and 4% stacking gels. Running buffer contained 192 mm glycine, 50 mm Tris-Cl, 0.1 mm CaCl_2_, pH 8.3. Separating gel buffer (2×) contained 0.7 m Tris-Cl and 1 mm CaCl_2_, pH 8.8. Stacking buffer (2×) contained 0.5 m Tris-Cl and 1 mm CaCl_2_, pH 6.7. Peptides were incubated with CaM for ≥1 h at 4 °C in gel filtration buffer (150 mm NaCl, 50 mm Tris-Cl, pH 7.5, 1 mm CaCl_2_) prior to loading. 50% glycerol was used in the gel filtration buffer, and <0.1% bromphenol blue (as tracker dye) was used at 1:2 sample volume just prior to loading for each sample. Gels were run at 100 V in 4 °C jacketed/ice-bath conditions for 6–8 h as needed. All gel-mobility-shift assays were confirmed in three replicate experiments.

### Isothermal titration calorimetry

Heat gain or heat requirements in the sample cell (for exothermic binding or endothermic binding, respectively) correspond to the direction of the injection peaks measured in the isothermal titration calorimeter (MicroCal iTC200, Malvern Instruments, UK). The peaks were integrated and plotted as a sigmoidal plot of μcal (concentration of the ligand in the cell) *versus* μmol of injectant (protein concentration in the syringe). Origin 2017 software (OriginLab) was used to fit the resulting data through iterative least-squares-type regression. Due to the tendency of CaMB peptides to aggregate in any salt, we reconstituted the CaMB peptides for ITC experiments in a low ionic strength (10 mm HEPES, pH 7.0, 0.1 mm CaCl_2_) buffer for all CaMB peptides. All experiments were performed at 25 °C, with 1000 rpm stirring, 5 μcal/s reference power, and high-feedback gain. Injection conditions were typically 1.2–1.8 μl each (22–28 injections), and cell/syringe concentrations were optimized for individual experiments (*c* value between 10 and 1000) (87–89) but typically between 10 and 50 μm for the cell and 100 to 500 μm for the syringe. Periodically, water-water and buffer-buffer controls were conducted to ensure the syringe and cell were clean and in good working order. Most of the endothermic titrations had to be baseline-subtracted against the final injection heats (end-weighted auto-baseline or manual baseline for Ca_v_3.3 correct for the heat of dilution) due to fitting artifacts from the isotherm passing through the *x* axis. Baseline-corrected curves were all fitted to a one-set-of-sites model in Origin 2017 (OriginLab) software with satisfactory results.

### Electron microscopy

Full-length mammalian Ca_v_3.1 was expressed in Sf9 insect cells and purified as described previously (38). Recombinant human Ca_v_3.1 was incubated (1:1 molar ratio) with biotin–CaM (Calbiochem) in 1.5 mm CaCl_2_ after which a streptavidin–gold conjugate (5 nm) was added (38). Samples were examined using standard negative staining techniques with 2% (w/v) uranyl acetate. Images of the Ca_v_3.1–CaM complexes were recorded on a Tecnai 12 transmission electron microscope operated at 100 kV.

### NMR

Complex formation of gating brake peptide with CaM in a 1:1 ratio was monitored by acquiring a ^1^H-^15^N heteronuclear single-quantum coherence (HSQC) spectrum in a manner previously published with CaM and peptides (90). NMR spectra were recorded at 25 °C on Bruker 600 and 700 MHz DRX spectrometers equipped with XYZ-gradient triple-resonance probes (Bruker, Billerica, MA), and analyzed with CARA (computer-aided resonance assignment). CaM for NMR experiments was expressed in M9 minimal media (11.03 g/liter Na_2_HPO_4_·7H_2_O, 3.0 g/liter KH_2_PO_4_, 0.5 g/liter NaCl, 2 mm MgSO_4_, 0.1 mm CaCl_2_, 5 mg/ml thiamine, 100 μg/ml kanamycin) containing 2 g/liter [^13^C]glucose and 1 g/liter ^15^NH_4_Cl; ^13^C-^15^N CaM was purified. The samples were prepared for NMR experiments via a buffer exchange into NMR solution (100 mm KCl, 10 mm CaCl_2_, 0.2 mm NaN_3_, 90% H_2_O, 10% 2H_2_O) at pH 6.0 using a YM10 centrifugal filter device (Millipore, Billerica). All NMR samples contained 300 μm CaM in a total volume of 500 μl. The samples were transferred into 5-mm NMR sample tubes and stored at 4 °C until required for NMR experiments. NMR experiments on the complexes were conducted on CaM samples titrated with each peptide to saturation in a 1:1 CaM/peptide ratio. Complex formation was monitored after each addition by acquisition of a ^1^H-^15^N HSQC pulse sequence. NMR spectra were recorded at 25 °C on Bruker 600 and 700 MHz DRX spectrometers equipped with XYZ-gradients triple-resonance probes (Bruker, Billerica, MA.). Spectra were analyzed using the program CARA.

### Electrophysiological recordings

Macroscopic currents were recorded at room temperature using an Axopatch 200B amplifier (Molecular Devices). Borosilicate glass pipettes had a resistance of 1.5–2.5 megaohms when filled with an internal solution containing (in mm): 140 CsCl, 10 EGTA, 10 HEPES, 3 Mg-ATP, 0.6 GTP-Na (pH adjusted to 7.25 with CsOH, ∼315 mosm). The extracellular solution contained (in mm): 145 tetraethylammonium-Cl, 10 HEPES, and 2 CaCl_2_ (pH adjusted to 7.25 with tetraethylammonium-OH, ∼330 mosm). Recordings were filtered at 2 kHz. Data were analyzed using pCLAMP9 (Molecular Devices), GraphPad Prism (GraphPad), and Origin 2017 (OriginLab) software. Current-voltage relationships were generated in 5- or 10-mV voltage steps from a holding potential of −100 mV. Steady-state inactivation curves were constructed by plotting normalized peak currents (peak test pulse current/peak pre-pulse current) as a function of inactivating potentials. Kinetics of activation and inactivation were determined by fitting mono-exponential functions over the growing or decaying phases of each current trace.

### Statistical analyses

Results are presented as mean ± S.E., and *n* = the number of cells. Statistical analysis was first performed with the Student's *t* test or with one-way ANOVA combined with a Tukey post-test for multiple comparisons (*, *p* < 0.05; **, *p* < 0.01; ***, *p* < 0.001). For the electrophysiology data sets as paired data of treatment values compared with control values at time 0 and after 20 min of CaMB peptide dialyses in Figs. 7 and 8, we also used a non-parametric Wilcoxon matched-pairs signed rank test. In Fig. 9, there were three sets of data to compare between wild type, CaM overexpression, and CaM_1234_ overexpression, so we used a Kruskal-Wallis test here followed by a Dunn's multiple comparison test or a Mann-Whitney test. The statistical analyses using parametric and non-parametric analyses produced similar results.

### Co-immunoprecipitation experiments

Forty eight hours after transfection, cells cultured in 35-mm dishes were lysed on ice for 20 min with Nonidet P-40 buffer containing 50 mm Tris-HCl, pH 7.4, 125 mm NaCl, 25 mm MgCl_2_, 33.3 μm CaCl_2_, 5% glycerol, 1% Nonidet P-40. Cell lysates were centrifuged at 10,000 × *g* for 30 min at 4 °C. Clarified cell lysates were then incubated with anti-GFP-conjugated magnetic beads (Thermo Fisher Scientific) for 4 h at 4 °C. The beads were washed four times with lysis buffer and resuspended in 2× loading buffer. The immunoprecipitates were then analyzed by SDS-PAGE and Western blotting.

### Western blotting

Samples were loaded on SDS-PAGE. Proteins were then transferred onto nitrocellulose membranes and blocked with 5% powdered nonfat milk in PBS-T (Tween 0.1%). Primary antibody was incubated overnight at 4 °C in 5% powdered nonfat milk in PBS-T (Tween 0.1%). After three washes in 5% powdered nonfat milk in PBS-T, the secondary HRP-coupled antibodies were incubated for 1 h at room temperature in the same buffer. The signal was detected using the Super Signal West Pico chemiluminescent system (Thermo Fisher Scientific).

### Antibodies and reagents

The antibodies used in the study were as follows: rat anti-HA (3F10, Roche Applied Science; 1:1000) and secondary goat anti-rat antibody coupled to horseradish peroxidase (112-036-072 Jackson ImmunoResearch; 1:5000); rabbit anti-GFP (TP401, Torrey Pines Biolabs; 1:5000); and secondary donkey anti-rabbit coupled to horseradish peroxidase (NA934 GE Healthcare; 1:10,000).

## Author contributions

The CaV3.1-baculovirus construct was made and Sf9 cells harvested in the laboratory of A. C. D., which were used for the single particular cryo-EM carried out in the laboratory of A. K. The co-immunoprecipitation experiments were carried out by A. M. in his laboratory. The gel-mobility-shift assays, circular-dichroism spectroscopy, and isothermal-titration calorimetry experiments were carried out by V. T. in the laboratory of J. G. G. Most electrophysiology experiments of mammalian Cav3.2 channels were conducted by J. C. in his laboratory, based on preliminary discoveries by the electrophysiology work of R. F. S. and W. G. in the laboratory of J. D. S. Cav3.2 channel mutants were provided by E. P. R., who was especially helpful in the design of electrophysiology experiments involving the gating brake. The NMR experiments were conducted by M. P. in the laboratory of T. D. Vectors for CaM1234 overexpression and shRNA knockdown of Calm1,2,3 genes were provided by Z. P. P. All authors contributed to the writing of the manuscript. Team management and the bulk of the writing of the manuscript was conducted by J. D. S.

## Footnotes

4 R. F. Stephens, W. Guan, O. Mourad, A. Mehta, and J. D. Spafford, manuscript in revision.

5 W. Guan, R. F. Stephens, and J. D. Spafford, manuscript in revision.

6 Please note that the JBC is not responsible for the long-term archiving and maintenance of this site or any other third party hosted site.

## Footnotes

3 The abbreviations used are:

CaM: calmodulin
aa: amino acid
BisTris: 2-[bis(2-hydroxyethyl)amino]-2-(hydroxymethyl)propane-1,3-diol
ITC: isothermal titration calorimetry
CaMB: CaM binding
ANOVA: analysis of variance
TFE: trifluoroethanol
BAPTA: 1,2-bis(2-aminophenoxy)ethane-*N*,*N*,*N*′,*N*′-tetraacetic acid
NSCaTE: N-terminal spatial Ca^2+^-transforming element
RFP: red fluorescent protein
HSQC: heteronuclear single-quantum coherence
dansyl: 5-(dimethylamino)naphthalene-1-sulfonyl chloride
CARA: computer-aided resonance assignment
ESI-MS: electrospray ionization-mass spectrometry
EGFP: enhanced GFP.
